# Characterization of Non‐Specific Electrostatic Interactions of Cationic Peptides with DNA Origami and Their Functional Consequences

**DOI:** 10.1002/smtd.202501936

**Published:** 2025-12-05

**Authors:** Seung Hyun Kang, Oheun Kwon, Bo Kyung Cho, Seungmin Yoo, Jin Myeong Wang, Youngjin Choi, Hong Yeol Yoon, Jungkyu Choi, Ju Hee Ryu

**Affiliations:** ^1^ Medicinal Materials Research Center Biomedical Research Institute Korea Institute of Science and Technology (KIST) Seoul 02792 Republic of Korea; ^2^ Department of Chemical and Biological Engineering Korea University Seoul 02481 Republic of Korea; ^3^ KU‐KIST Graduate School of Converging Science and Technology Korea University Seoul 02841 Republic of Korea

**Keywords:** cationic peptide, DNA origami, electrostatic interaction, non‐specific binding, stoichiometric control

## Abstract

The functionalization of DNA origami with peptides is a powerful strategy for creating nanodevices for therapeutic and diagnostic applications. A critical but often overlooked challenge is the non‐specific electrostatic binding of cationic peptides to the anionic DNA nanostructure, which leads to uncontrolled stoichiometry and undermines functional predictability. Here, the study systematically characterizes this issue and demonstrates a practical purification strategy to mitigate it. It is quantitatively shown that cationic peptides associate with DNA origami in vast excess of their intended binding sites, a phenomenon not observed with anionic control peptides. This non‐specific binding is confirmed to be electrostatic and is effectively screened by high salt. To address this, a charge‐dependent purification approach is evaluated using polyethylene glycol (PEG) precipitation, showing that cationic peptides require extensive purification (≥7 cycles), whereas anionic peptides need only minimal treatment (2 cycles) to achieve precise loading. Crucially, the study provides definitive functional evidence that a therapeutic peptide (brain‐derived neurotrophic factor‐mimicking peptide) must be attached via stable, site‐specific hybridization to elicit a potent biological response; non‐specifically adsorbed peptides are largely inactive. This work provides a set of critical design guidelines and purification considerations necessary for the rational design of reliable and functionally predictable DNA nanodevices.

## Graphical abstract

DNA origami offers a precise platform for peptide functionalization, but its negatively charged scaffold causes severe non‐specific adsorption of cationic peptides. This study systematically quantifies this electrostatic artifact and introduces a charge‐tailored polyethylene glycol (PEG) purification workflow that restores stoichiometric control. The authors further demonstrate that only site‐specifically hybridized therapeutic peptides retain biological activity, guiding reproducible functional DNA nanodevice design.

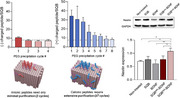

## 1 Introduction

DNA nanotechnology, particularly the DNA origami technique, enables the bottom‐up assembly of nanostructures with precisely defined, custom geometries.^[^ ^1^, ^2^ ^]^ A key application of this precision is the functionalization of these nanostructures with ligands–functional molecules such as proteins, peptides, or small molecules–to create advanced therapeutic and diagnostic devices.^[^ ^3^, ^4^ ^]^ The power of this approach lies not merely in attaching these ligands, but in the site‐specific control over their number (stoichiometry) and nanoscale spatial organization. It is now well‐established that this precise arrangement of ligands is a critical determinant of biological function, directly influencing outcomes like receptor activation and immune cell signaling.^[^ ^3^, ^5^, ^6^, ^7^, ^8^, ^9^ ^]^ Therefore, achieving this level of stoichiometric and spatial control is the fundamental goal when designing functional DNA nanodevices.

Beyond sequence‐programmed functionalization, cationic coatings—including polymers, proteins/peptides, and other cationic nanomaterials—have been widely employed on DNA origami.^[^ ^10^, ^11^, ^12^, ^13^ ^]^ Such coatings predominantly arise from non‐specific electrostatic adsorption onto the polyanionic phosphate backbone and modulated by the ionic environment. Because this mode of association lacks spatial control, it is conceptually distinct from site‐specific hybridization. Building on this context, we focus here on cationic peptide–DNA origami interactions and quantitatively contrast non‐specific peptide adsorption with site‐specific placement.

Among the various types of molecular cargo, peptides are particularly powerful, as they can mimic the specific biological function of much larger proteins with a low molecular weight.^[^ ^14^, ^15^, ^16^ ^]^ Peptides can be designed to have a wide range of physicochemical properties, including a net anionic, neutral, or cationic charge. While each class has its utility, it is the cationic peptides that present a fundamental and often underestimated challenge in the context of DNA nanotechnology.^[^ ^17^, ^18^, ^19^, ^20^ ^]^ The strong, non‐specific electrostatic attraction between these positively charged peptides and the highly anionic DNA framework leads to uncontrolled, non‐stoichiometric loading, which compromises the precision and function of the final nanodevice.^[^ ^21^ ^]^

While the non‐specific binding of cationic peptides is a known phenomenon, it is rarely systematically quantified, and its impact on purification and final device function is not well understood. This lack of systematic characterization can lead to the production of heterogeneous materials with an unknown number of active ligands, confounding the interpretation of biological data and hindering the development of reliable nanotechnologies. There is a clear need for a robust framework to characterize, control, and mitigate these non‐specific interactions.

Here, we provide a comprehensive characterization of the non‐specific binding of peptides to DNA origami, focusing on the role of peptide charge. We first demonstrate that cationic peptides bind in vast, multi‐fold excess of their intended binding sites, a problem not observed with anionic peptides. We then establish polyethylene glycol (PEG) precipitation as a practical strategy to purify these complexes, quantifying the number of purification cycles required to achieve precise stoichiometric control. Finally, we provide functional evidence that this rigorous control over peptide attachment is not just a matter of analytical precision but is absolutely essential for the biological activity of the final nanostructure, offering a critical set of design guidelines.

## 2 Results and Discussion

### 2.1 Synthesis and Characterization of a Diverse Peptide‐ssDNA Panel

To investigate the effect of peptide charge on DNA nanostructure assembly, we first synthesized a series of peptide‐ssDNA conjugates. This panel was designed to cover a wide range of physicochemical properties and includes cationic (CP), anionic (AP), and neutral (NP) peptides with net charges ranging from +5 to −4. The detailed properties of each peptide, including sequence, are summarized in **Table** **1**.

**Table 1:** Physicochemical properties and systematic names of the peptide panel.

Peptide ID	Peptide sequence N→C	Length	Net charge [pH 7]^a^ ^)^	MW [g mol^−1^]^b^ ^)^	pI^b^ ^)^	Conjugation buffer^c^ ^)^	Conjugation yield [%]^d^ ^)^	Refs.
CP1	YRSRKYSSWYVALKR	15	+5	1963.3	10.9	A	57.1	[22]
CP2	GGGSEEEYRSRKYSSWYVALKR	22	+2	2608.9	9.8	A	79.3	[22]
CP3	RGIDKRHWNSQ	11	+2	1395.7	11.4	B	79.4	[23]
CP4	CRPGWRGAACNQKIL	15	+3	1672.0	10.2	A	80.2	[24]
CP5	PKKKR	5	+3*	655.0	11.8	B	85.9	[14]
CP6	KLTWQELYQLKYKGI	15	+2	1910.1	9.9	B	51.5	[16]
CP7	CLQKTPKQC	9	+2	1048.3	8.9	B	88.8	[25]
CP8	NYSKPTDRQYHF	12	+1	1555.7	8.5	B	70.9	[26]
CP9	KVPRNQDWL	9	+1	1155.0	10.1	A	79.7	[27]
AP1	PSKPSFQEFVDWENVSPELNSTDQPFL	27	−4	3136.0	3.5	A	45.3	[28]
AP2	EQLESIINFEKLTEWT	16	−3	1980.2	3.8	A	76.4	[29]
AP3	ISQAVHAAHAEINEAGR	17	−1	1774.0	6.0	A	76.9	[30]
NP1	REGVELCPGNKYEMRRHGTTHSLVIHD	27	0	3134.0	7.4	B	81.7	[28]
NP2	GGGG	4	0	246.1	6.0	C	89.1	[31]
NP3	AAAA	4	0	302.2	6.0	C	90.6	[31]
NP4	LLLL	4	0	470.3	6.0	C	68.4	[32]
NP5	SSSHPIFHRGEFSV	14	0	1585.8	8.0	B	39.0	[15]

^a)^ Net charges were calculated at pH 7.0 using the PepCalc tool (pepcalc.com). For the cyclic peptide CP5, the effective net charge is +3 rather than the theoretical +4, as one lysine was modified for azide conjugation;

^b)^ Molecular weight (MW) and isoelectric point (pI) values were calculated using the Thermo Scientific Peptide Analyzing Tool;

^c)^ Buffers used were as follows: A, 7% DMSO, 9.3 mm Tris‐HCl, 0.93 mm EDTA; B, 10 mm Tris‐HCl, 1 mm EDTA, pH 8.0; C, 90% DMSO, 1 mm Tris‐HCl, 0.1 mm EDTA;

^d)^ Conjugation yields were determined from mobility shifts in denaturing polyacrylamide gel electrophoresis (dPAGE). Band intensities were quantified using ImageJ, and the yield was defined as the ratio of the conjugate band intensity to the total intensity of all DNA‐containing bands in the same lane.

**Figure 1:**
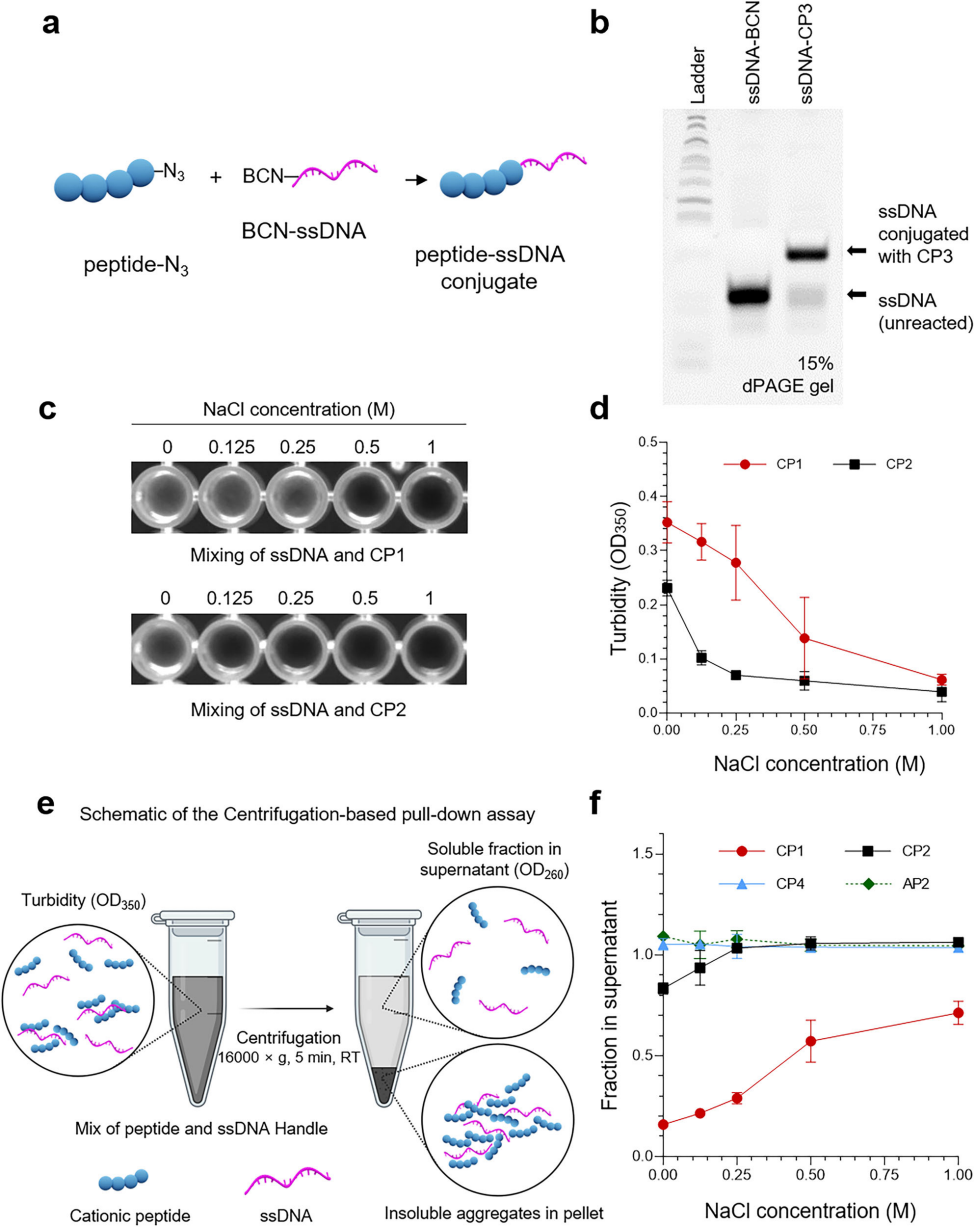
Aggregation of peptide‐ssDNA conjugates is driven by cationic charge and is highly salt‐sensitive. a) Schematic of the strain‐promoted azide‐alkyne cycloaddition (SPAAC) reaction used to generate peptide‐ssDNA conjugates. b) Representative 15% denaturing polyacrylamide gel electrophoresis (dPAGE) confirming successful conjugation for the peptide CP3. The conjugated product exhibits a clear mobility shift compared to the unreacted ssDNA starting material. c) Visual turbidity of mixtures containing ssDNA and the cationic peptides CP1 (top) or CP2 (bottom) in a 96‐well plate, shown as a function of increasing NaCl concentration. d) Quantification of the turbidity from (c) by measuring the optical density at 350 nm (OD_350_). Data are presented as mean ± SD from n = 3 independent replicates. e) Schematic of the centrifugation‐based pull‐down assay used to quantify peptide‐ssDNA interactions. In this assay, peptide‐ssDNA mixtures are centrifuged to separate large, insoluble aggregates (which form a pellet) from the soluble, unbound ssDNA (which remains in the supernatant). The amount of ssDNA in the supernatant is then quantified to determine the extent of the interaction. f) Salt‐dependence of the soluble ssDNA fraction remaining in the supernatant after centrifugation with various peptides. The aggregation‐prone cationic peptides CP1 and CP2 are compared against the non‐aggregating cationic peptide CP4 and anionic peptide AP2. Data are presented as mean ± SD (*n* = 3).

Each peptide was conjugated to a bicyclo[6.1.0]nonyne (BCN)‐modified ssDNA (ssDNA 1; sequence shown in Table S1, Supporting Information) via strain‐promoted azide‐alkyne cycloaddition (SPAAC), as depicted in **Figure** **1**a. Successful conjugation for all peptides was confirmed by denaturing polyacrylamide gel electrophoresis (dPAGE). As shown in the representative gel in Figure 1b and the comprehensive panel in Figure S1 (Supporting Information), each conjugated product exhibited a distinct electrophoretic mobility shift compared to the unreacted ssDNA, consistent with an increase in molecular weight. The conjugation yields for this panel, ranging from 39.0% to 90.6% are summarized in Table 1. These yield data were included primarily to validate successful and reproducible synthesis of the peptide‐ssDNA conjugates for their use in downstream DNA origami functionalization. This chemical yield should be considered distinct from the physical charge‐driven interactions (e.g., aggregation) of the peptides, which are examined separately in the following analyses.

Upon mixing BCN‐modified ssDNA with azide‐functionalized peptides for conjugation in low‐salt buffers, we observed that several of the highly cationic peptides underwent aggregation, which manifested as solution turbidity (Figure S2a, Supporting Information). The persistence of this turbidity varied by peptide: CP1 and CP2 formed stable aggregates, whereas CP3 and CP4 produced only transient cloudiness. In addition, wells containing peptides alone showed no visible turbidity (Figure S2c, Supporting Information), further confirming that the observed turbidity originates from peptide‐DNA co‐aggregation rather than peptide self‐precipitation. To confirm that this aggregation was an intrinsic property of the peptide‐ssDNA interaction—rather than an artifact of the BCN moiety or the conjugation chemistry itself—we performed a control experiment by mixing the peptides with unmodified ssDNA lacking the BCN group. We observed an identical pattern of turbidity (Figure S2b, Supporting Information), confirming that the aggregation is driven by electrostatic interactions between the cationic peptides and the anionic ssDNA backbone.^[^ ^33^ ^]^

Controlling this intrinsic aggregation is critical, as the formation of such insoluble aggregates can lead to loss of biological function and prevent accurate quantification.^[^ ^34^, ^35^ ^]^ We therefore focused on the most strongly aggregating peptides, CP1 and CP2, examining their behavior under controlled salt titration. The addition of NaCl resulted in a dose‐dependent clearing of the solution's turbidity. This effect was quantified by a decrease in optical density at 350 nm (OD_350_), a method to measure turbidity arising from light scattering by macromolecular aggregates (Figure 1c,d).^[^ ^36^, ^37^ ^]^ This result confirms that the aggregation is driven by electrostatic interactions.^[^ ^38^, ^39^ ^]^

Next, to quantify the propensity of these peptides to induce the aggregation, we developed a centrifugation‐based pull‐down assay (Figure 1e). In this assay, the cationic peptide pulls down the interacting BCN‐free ssDNA, forming large macromolecular aggregates that are sedimented as a pellet. The amount of ssDNA remaining in the soluble supernatant is then determined by measuring its absorbance at 260 nm.^[^ ^40^, ^41^ ^]^ As shown in Figure 1f, the aggregation‐prone cationic peptides CP1 and CP2 both exhibited salt‐dependent solubility. The soluble fraction for both peptides increased as the NaCl concentration was raised, confirming that the aggregation is driven by electrostatic interactions. In contrast, the cationic peptide CP4 behaved differently, remaining highly soluble across all conditions, similar to the anionic control peptide AP2. These results demonstrate that while a high positive charge can be a strong driver for aggregation, this behavior is not universal among all cationic peptides and is dependent on other specific physicochemical properties of the peptide.^[^ ^33^ ^]^

Notably, the extent of aggregation observed in this study did not correlate with the conjugation yields summarized in Table 1. This apparent independence can be explained by two complementary considerations supported by prior findings. First, differences in conjugation yield across the peptide panel are likely influenced by sequence‐specific physicochemical features—such as steric accessibility, hydrophobicity, or local electrostatics near the reactive site—which can affect the effective exposure of functional groups.^[^ ^42^, ^43^, ^44^ ^]^ As prior literature confirms, conjugation yield is a highly complex outcome, and it is reasonable that aggregation and yield did not show a direct correlation in this study. Second, the SPAAC reaction is inherently robust and known to tolerate diverse microenvironments, including crowded or partially aggregated systems.^[^ ^45^, ^46^ ^]^ Such robustness suggests that moderate aggregation or local heterogeneity may have only limited impact on SPAAC reactivity.^[^ ^42^, ^45^, ^46^ ^]^

To further examine this relationship, additional pull‐down assays were conducted using six additional peptides (CP3, CP5, CP6, AP3, NP1, and NP5) that span diverse charge, solubility, and structural characteristics (Figure S3, Supporting Information). These experiments confirmed that aggregation extent did not correlate with the conjugation yields, and highlight that further investigation into these physicochemical aspects will be valuable to fully understand peptide‐dependent conjugation efficiency. Importantly, the aggregation assays in this study were not intended to explain yield variance but to establish a charge‐mediated interaction framework that underlies the subsequent peptide–DNA origami complexation analyzed in later sections.

### 2.2 Characterization of Charge‐Dependent Non‐Specific Binding to DNA Origami

**Figure 2:**
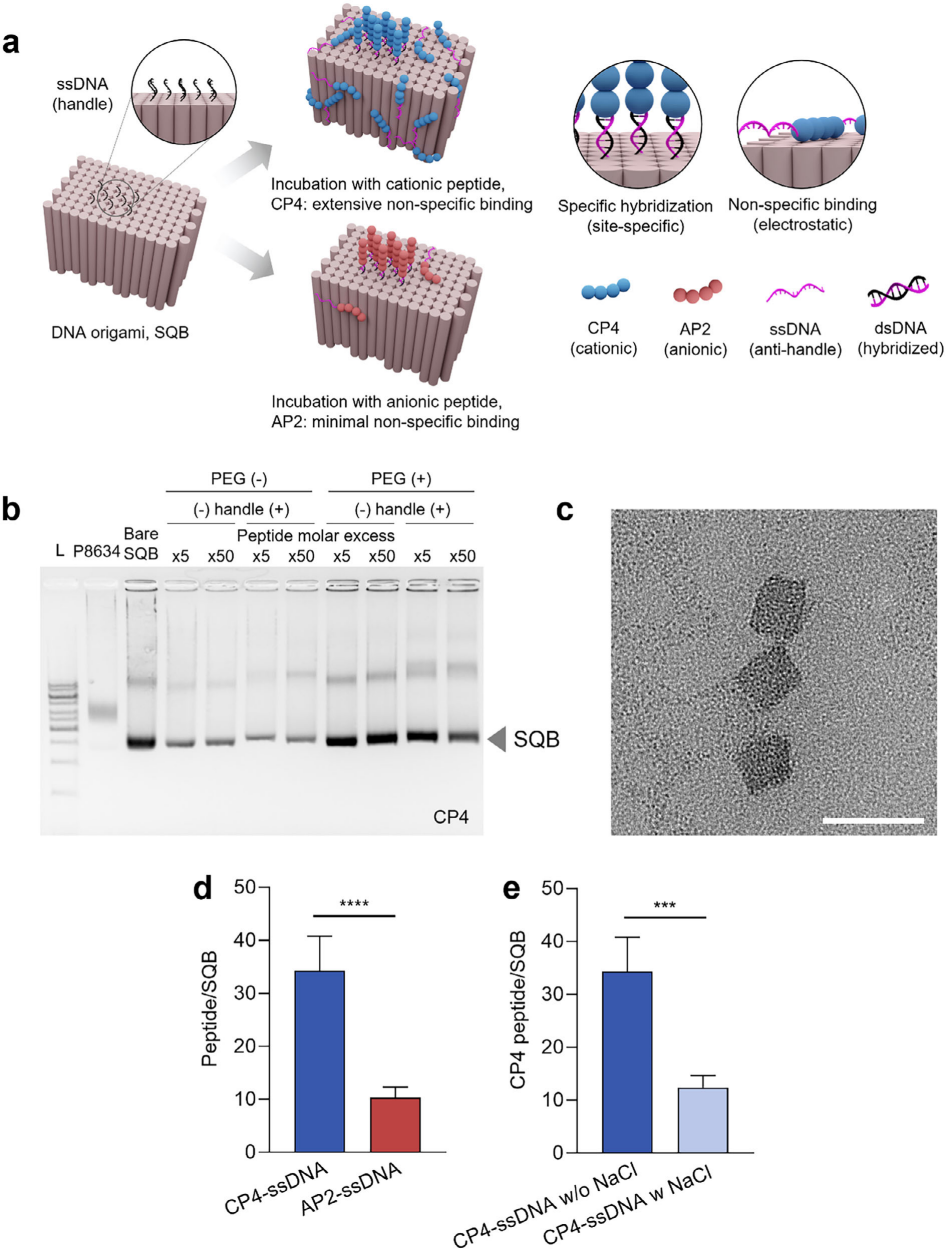
Charge‐dependent non‐specific binding of peptides to the DNA origami square block (SQB). a) Schematic illustrating charge‐dependent peptide functionalization. Cationic peptides (CP4, blue) exhibit extensive non‐specific electrostatic adsorption to the anionic SQB surface, while anionic peptides (AP2, red) show minimal non‐specific binding, primarily attaching via site‐specific hybridization. Right panel provides a detailed legend for all molecular components. b) Agarose gel electrophoresis confirming successful handle/anti‐handle hybridization. A clear upward band shift is observed for the SQB incubated with the CP4‐conjugate [handle (+)] relative to the bare SQB. c) Representative transmission electron microscopy (TEM) image of CP4‐conjugated SQBs, confirming the preservation of the intended square‐block morphology. Scale bar, 50 nm. d) Quantitative comparison of peptide loading on SQBs after incubation with a fivefold molar excess of either cationic ssDNA‐CP4 or anionic ssDNA‐AP2, as measured by high‐performance liquid chromatography (HPLC). e) Quantification of non‐specific binding for the cationic ssDNA‐CP4 conjugate in the presence or absence of a high‐salt (1 m NaCl) buffer, demonstrating that the interaction is electrostatic in nature. All bar graphs show mean ± SD (*n* = 4–5). Statistical analysis was performed using an unpaired *t*‐test; ****p < 0.0001, ***p < 0.001.

To functionalize our DNA origami square block (SQB) with peptides, we aimed to attach a precise number of peptide‐ssDNA conjugates to the nanostructure. This attachment is mediated by site‐specific hybridization between two complementary ssDNA sequences: “handles”, which are pre‐designed into the DNA origami, and “anti‐handles”, which are the ssDNA component of the peptide‐ssDNA conjugates (**Figure** **2**a). Successful handle/anti‐handle hybridization was verified by a clear band shift in agarose gel electrophoresis (AGE) for the cationic peptide CP4‐conjugated SQB relative to the bare SQB (Figure 2b), with similar confirmations for the anionic AP2 and neutral NP1 conjugates (Figure S4a, Supporting Information). Transmission electron microscopy (TEM) further confirmed that the SQB architecture was preserved after conjugation with CP4, AP2, and NP1, with no discernible deformation or aggregation (Figure 2c; Figure S4b, Supporting Information).

We hypothesized that the net charge of the peptide would critically influence this process, with cationic peptides potentially causing significant non‐specific binding to the anionic DNA origami.^[^ ^47^ ^]^ To investigate this, we designed SQBs with eight specific binding sites and quantified the number of attached peptides after incubation with a 5‐fold molar excess of either the cationic CP4‐ssDNA (+3) conjugate or the anionic AP2‐ssDNA (−3) conjugate. Our quantitative reverse‐phase high‐performance liquid chromatography (RP‐HPLC) analysis, performed after DNase digestion of the SQB, revealed a dramatic, charge‐dependent difference in peptide loading. AP2 and CP4 were quantified by RP‐HPLC, and their identities were confirmed by matching retention times with authentic standards (Figure S5a–d, Supporting Information). For the cationic CP4‐ssDNA, we detected an average of ≈35 peptides per SQB (Figure 2d). This is ≈4.4 times higher than the eight intended binding sites, indicating that ≈27 peptides were non‐specifically attached via electrostatic interactions. In contrast, the anionic AP2‐ssDNA resulted in the attachment of ≈10 peptides per SQB, indicating only minimal non‐specific binding.

To confirm that this pronounced non‐specific binding was electrostatically driven, we repeated the CP4 incubation in a high‐salt buffer containing NaCl. Agarose gel electrophoresis and transmission electron microscopy confirmed that the SQB remained structurally stable even at 1 m NaCl, indicating that the high‐salt condition did not compromise particle integrity (Figure S6a,b, Supporting Information). As expected, the presence of salt drastically reduced the number of bound CP4‐ssDNA conjugates from 34.3 ± 5.6 to 12.4 ± 2.0 (Figure 2e; Figure S7, Supporting Information). Collectively, these results demonstrate that non‐specific binding is a significant, charge‐dependent challenge for cationic peptides, and that this interaction is fundamentally electrostatic in nature.

### 2.3 Stoichiometric Control of Peptide Loading via Optimized PEG Precipitation

**Figure 3:**
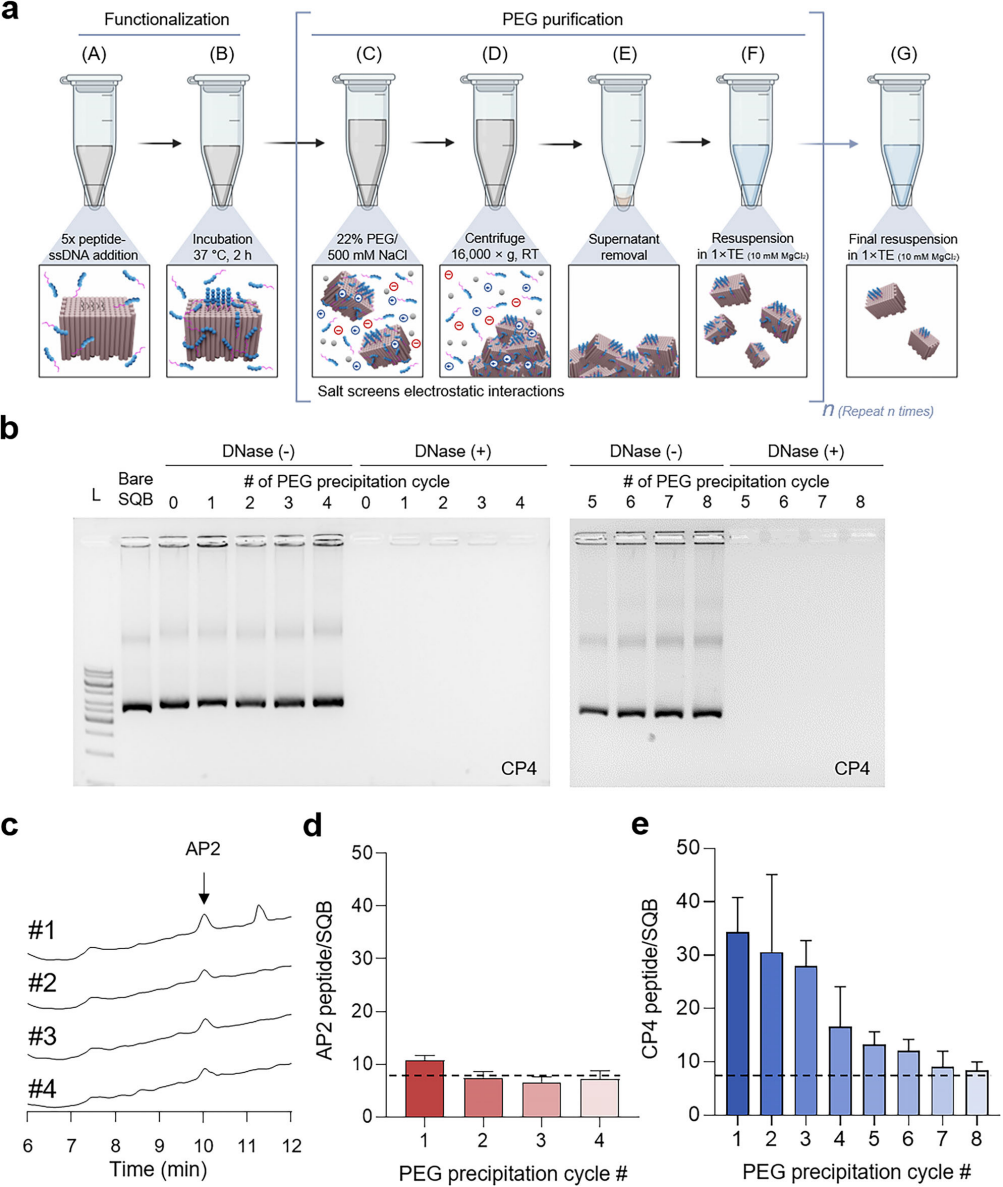
Charge‐dependent purification of peptide‐conjugated SQB via repeated PEG precipitation. a) Schematic of the functionalization and iterative PEG purification process. Bare SQBs are first functionalized via hybridization with peptide‐ssDNA conjugates, followed by *n* cycles of PEG/salt‐induced precipitation to remove unbound and non‐specifically adsorbed peptides. b) Agarose gel electrophoresis tracking the structural integrity of the cationic CP4‐conjugated SQB through eight PEG precipitation cycles. Samples were analyzed with [DNase(+)] or without [DNase(−)] nuclease treatment to confirm the structure's integrity and susceptibility to degradation, respectively. c) Representative HPLC chromatograms showing the amount of the anionic AP2 peptide remaining after purification cycles #1 to #4. d) Quantification of AP2 peptides per SQB versus PEG precipitation cycle, derived from the HPLC data in (c). e) Quantification of cationic CP4 peptides per SQB versus PEG precipitation cycle, demonstrating the requirement of extensive purification. For all bar graphs, data are presented as mean ± SD.

Given that cationic peptides exhibit severe non‐specific binding, we next sought to implement and quantify a purification strategy to achieve precise stoichiometric control. We hypothesized that repeated PEG precipitation, a well‐established method widely used to purify DNA origami, could remove the weakly, electrostatically bound excess peptides, and that the optimal number of purification cycles would be highly dependent on the peptide's charge (**Figure** **3**a). This method is effective because PEG‐induced precipitation is strongly modulated by electrostatic interactions. The inclusion of salt (e.g., ≈500 mm NaCl) in the precipitation buffer screens charge repulsion between origami structures, facilitating their precipitation.^[^ ^48^, ^49^, ^50^, ^51^ ^]^ We reasoned that this same salt‐mediated charge screening would also weaken the non‐specific electrostatic attraction between cationic peptides and the DNA surface. Therefore, repeated PEG precipitation cycles under these conditions should selectively remove the non‐specifically adsorbed peptides while retaining the stably hybridized ones.

To test this, we compared the purification profiles of SQBs decorated with either the anionic AP2‐ssDNA or the cationic CP4‐ssDNA. First, we confirmed that the purification process did not compromise the structural integrity of the nanostructures. Agarose gel analysis showed that the SQB structures remained intact, appearing as a distinct band even after eight precipitation cycles (Figure 3b). Furthermore, these bands were shown to be fully susceptible to DNase I digestion, a necessary pre‐treatment step for the subsequent quantification of conjugated peptides by HPLC. With the structural integrity confirmed, we quantified the peptide loading after each PEG cycle via HPLC. The results revealed a pronounced, charge‐dependent difference. The anionic AP2‐conjugated SQB required minimal purification; the intended stoichiometry of approximately eight peptides was achieved after only two cycles (Figure 3c,d). In contrast, the cationic CP4‐conjugated SQB required an extensive protocol. The number of associated peptides decreased progressively with each cycle, and as many as seven to eight cycles were necessary to remove the non‐specifically bound excess and reach the target stoichiometry (Figure 3e; Figure S8, Supporting Information).

Together, these results provide a critical design principle for functionalizing DNA nanostructures: the purification strategy must be tailored to the charge of the cargo. While anionic cargos can be incorporated with high fidelity using minimal purification, cationic cargos like CP4 necessitate a stringent and repeated purification protocol to remove a significant number of non‐specifically bound molecules and achieve the desired stoichiometry.

### 2.4 Physicochemical Characterization of Peptide‐Functionalized SQBs by DLS

We next sought to characterize how peptide functionalization alters physicochemical properties of the purified SQBs using dynamic light scattering (DLS). The structural integrity of the samples used for DLS analysis, including the bare SQB, CP4‐conjugated SQB (SQB‐CP4), and AP2‐conjugated SQB (SQB‐AP2), were first confirmed by agarose gel electrophoresis (Figure S9a, Supporting Information). DLS analysis of the bare SQB showed a relatively monodisperse population with a consistent hydrodynamic size (Figure S9b, Supporting Information). However, upon functionalization with either the cationic CP4 or the anionic AP2 peptide, the samples exhibited a notable increase in polydispersity (Figure S9c,d, Supporting Information). This increased heterogeneity and batch‐to‐batch variability suggest that while DLS can indicate changes upon peptide conjugation, it may not be a sufficiently reliable method for precise, quantitative characterization of these specific, non‐spherical DNA origami conjugates under our experimental conditions.^[^ ^52^ ^]^

### 2.5 Peptide Charge Does Not Determine Cellular Uptake of Peptide‐SQBs

Having established a robust purification protocol, we next investigated whether the net charge of the surface‐displayed peptide influences the cellular internalization of SQBs. To assess cellular uptake, SQBs were functionalized on one face with peptides of different charges via complementary handle/anti‐handle hybridization, allowing the precise attachment of the cationic CP5 (+3), neutral NP5 (0), or anionic AP2 (–3) peptide, while the opposite face was labeled with FITC. First, ssDNA “anti‐handles” were synthesized with either FITC (FITC‐ssDNA1) or peptide (e.g., CP5‐ssDNA3), and their successful synthesis was confirmed by dPAGE (Figure S10a,b, Supporting Information). These conjugates were then hybridized to their complementary “handles” on the SQB. Prior to all in vitro cell experiments, SQBs were coated with K_10_‐PEG_5k_ to enhance their stability.

**Figure 4:**
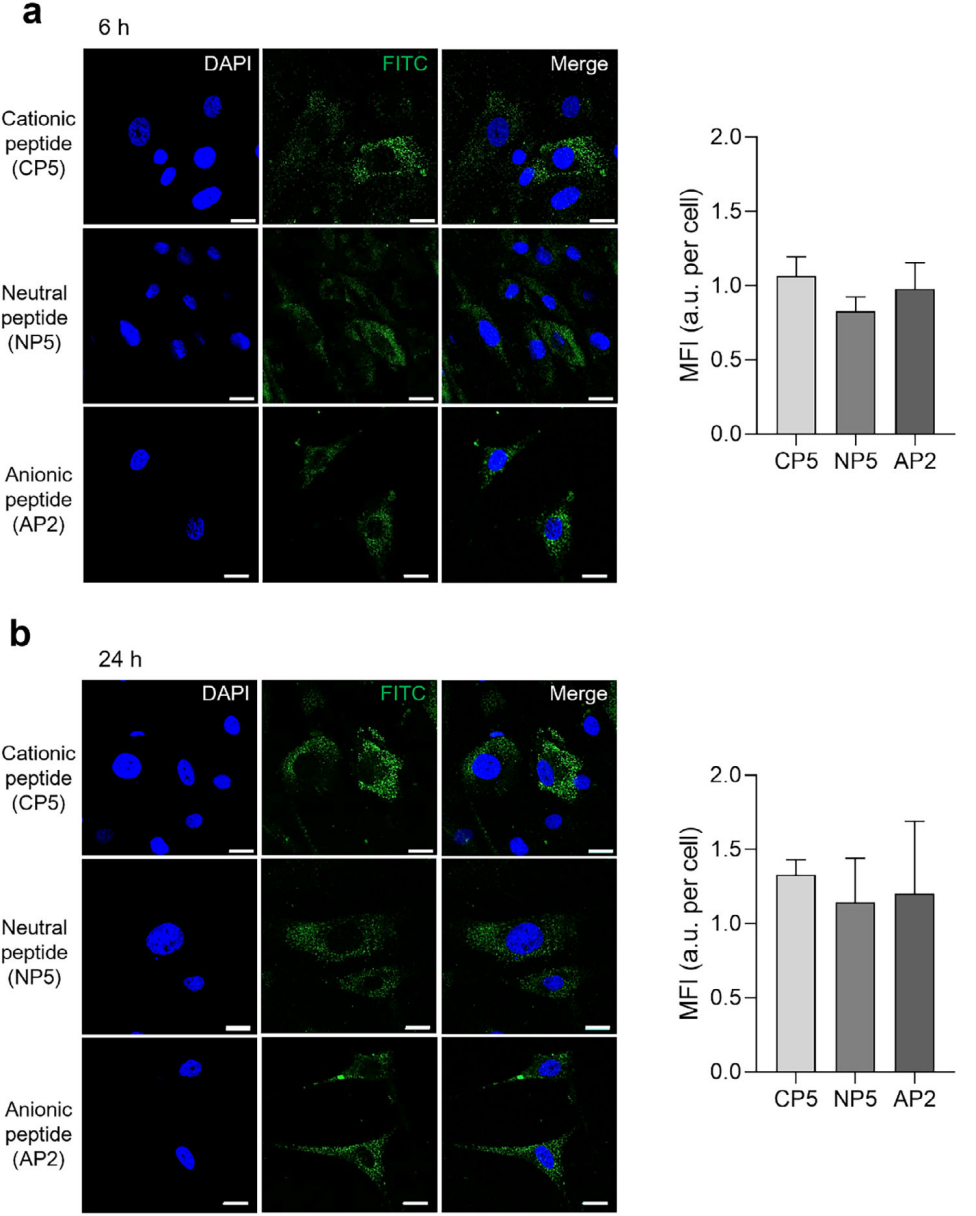
Peptide charge‐independent cellular uptake of SQBs. Representative confocal images and corresponding quantification of the cellular uptake of peptide‐functionalized SQBs in human mesenchymal stem cells (hMSCs) after a) 6 h and b) 24 h of incubation. For each time point, the left panels show confocal images of cells treated with SQBs functionalized with cationic (CP5), neutral (NP5), or anionic (AP2) peptides. SQBs were visualized by FITC fluorescence (green), and nuclei were stained with DAPI (blue). The right panels show the corresponding quantification of mean fluorescence intensity (MFI) per cell, calculated as total cytoplasmic FITC intensity normalized to the number of nuclei. No significant differences in uptake were observed among the three peptide charges at either time point, indicating that internalization is largely independent of the net charge of the conjugated peptide. Data are presented as mean ± SD from five randomly selected images per group. Statistical significance was analyzed by one‐way ANOVA. Scale bars, 20 µm.

Human mesenchymal stem cells (hMSCs) were treated with these peptide‐functionalized, FITC‐labeled SQBs. Confocal microscopy imaging at 6 and 24 h revealed efficient internalization across all three variants (**Figure** **4**a,b). Quantitative analysis of cellular uptake confirmed no significant differences in the uptake levels among the three peptide charges. These results indicate that the cellular uptake of DNA origami nanostructures is robust and largely independent of the net charge of the conjugated peptide.

To further investigate the effect of peptide presence, we performed a separate control experiment comparing peptide‐free SQBs with cationic peptide‐functionalized SQBs (SQB‐CP5). For this comparison, both constructs were labeled with Cy5.5 (using Cy5.5‐ssDNA2) for tracking. This control experiment revealed a significant increase in intracellular fluorescence for the peptide‐functionalized SQBs after 6 h incubation (Figure S11a,b, Supporting Information). These supplementary results suggest that while cellular uptake is largely independent of peptide charge (as shown in Figure 4), the presence of surface peptides enhances overall cellular uptake.

### 2.6 Synchronized Co‐Delivery Demonstrated with Dual Fluorophore Reporters on a Single DNA Origami

**Figure 5:**
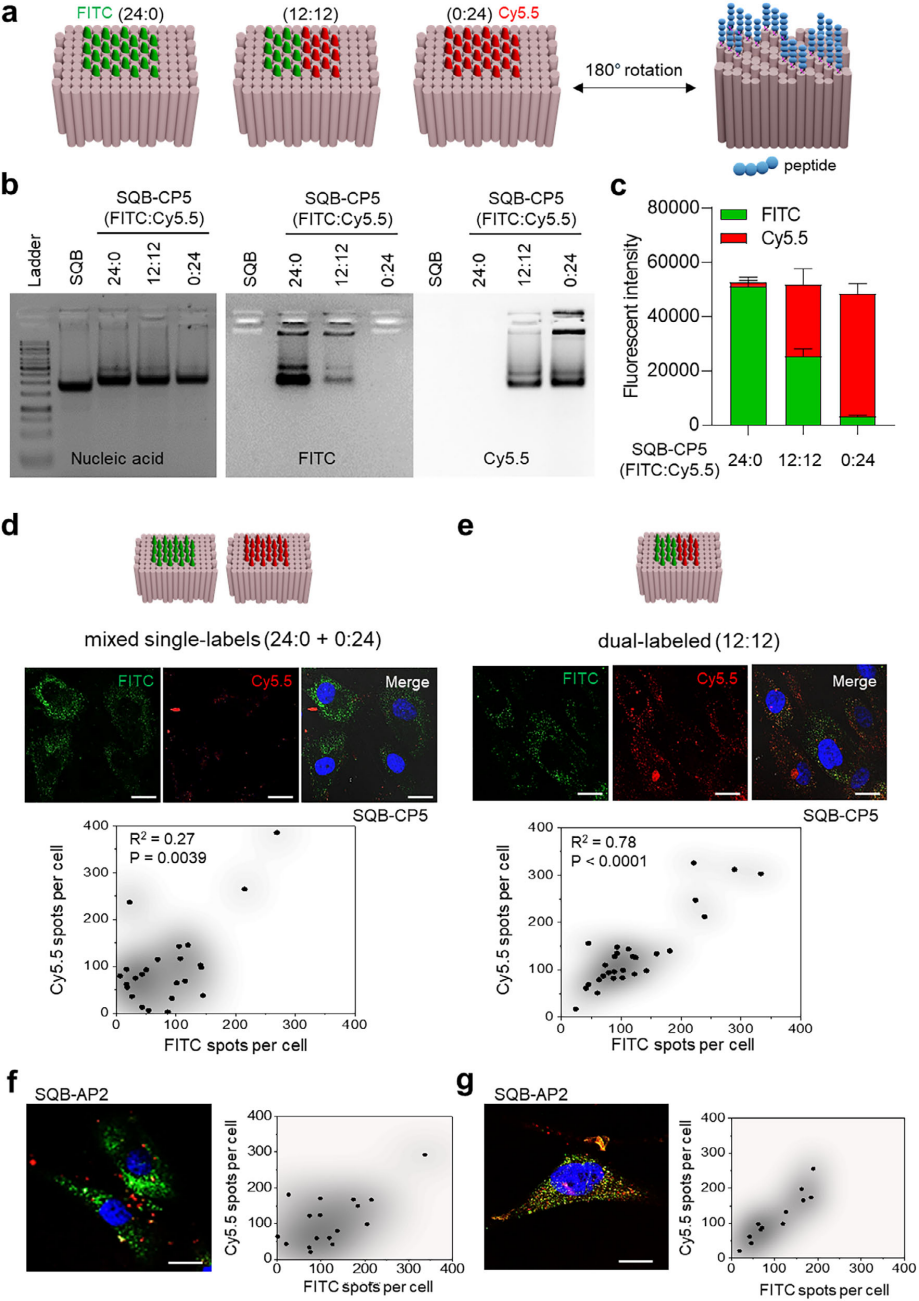
A Single DNA Origami Enables Synchronized Co‐delivery of Fluorophore Reporters. a) Schematic of the preparation of SQBs displaying fluorophores (FITC and Cy5.5) on the “even” face and a peptide on the opposite “uneven” face. b) Representative agarose gel confirming successful incorporation of FITC and Cy5.5 dyes onto the SQB‐CP5 scaffold at the indicated ratios. Gels were imaged for nucleic acid (left panel, stained with SYBR Gold) and in‐gel fluorescence (middle: FITC channel; right: Cy5.5 channel), confirming co‐localization of the dyes with the DNA origami bands. c) Quantification of fluorescence intensity from the gels, demonstrating precise stoichiometric control over the dye ratio. d–g) Confocal microscopy images and corresponding single‐cell correlation analyses comparing two delivery strategies: a 1:1 mixture of singly labeled SQBs (“mixed single‐labels”) versus a single population of dual‐labeled SQBs. The comparison was performed for SQBs functionalized with the cationic peptide CP5 (d,e) and the anionic peptide AP2 (f,g). For both peptide types, the dual‐labeled strategy resulted in a near 1:1 relationship between FITC and Cy5.5 signals (best‐fit slope 0.90 ± 0.09), indicative of true synchronized co‐delivery, whereas the mixed single‐label strategy showed a lower slope (0.72 ± 0.23). R^2^ and *p*‐values are provided as supporting metrics of correlation strength. Correlation plots show FITC spots per cell (x‐axis) versus Cy5.5 spots per cell (y‐axis). All images were taken after 6 h of incubation. Scale bars, 20 µm.

Having established a robust methodology for preparing stoichiometrically defined SQBs, we next sought to demonstrate a key advantage of the DNA origami platform: its ability to ensure the synchronized co‐delivery of multiple, distinct ligands. To model this capability, we first prepared SQBs displaying two different fluorophores, FITC and Cy5.5, on one face, and the cationic (CP5), anionic (AP2), or neutral (NP5) peptide on the opposite face (**Figure** **5**a). This was achieved by first synthesizing ssDNA “anti‐handles” covalently conjugated to either a fluorescent dye (FITC‐ssDNA1 or Cy5.5‐ssDNA2) or a peptide (e.g., CP5‐ssDNA3), each of which was confirmed to be pure by dPAGE (Figure S10a,b, Supporting Information). These conjugates were then hybridized to their complementary “handles” on the SQB. We confirmed that the two dyes were successfully incorporated onto the various peptide‐functionalized SQBs at the intended stoichiometric ratios (24:0, 12:12, and 0:24), using agarose gel analysis and in‐gel fluorescence imaging (Figure 5b,c).

Using these dye‐labeled nanostructures, we then validated the co‐delivery concept. We compared two delivery scenarios using confocal microscopy: a 1:1 mixture of two singly labeled SQB populations (mixed single‐labels; one bearing 24 FITC dyes, the other 24 Cy5.5 dyes) versus a single population of dual‐labeled SQBs, where each origami carried both 12 FITC and 12 Cy5.5 dyes.

Confocal microscopy and subsequent quantitative analysis revealed a significant, time‐dependent difference between the two delivery strategies. In the mixed single‐labels group, the cellular uptake of the two distinct SQB populations was clearly stochastic. After 6 h, this reflected in a relatively low best‐fit slope (0.72 ± 0.23) and a weak correlation with an R‐squared (R^2^) value (R^2^ = 0.27) between the two signals (Figure 5d,e). By 24 h, while the slope increased to 0.86 ± 0.18 and R^2^ value increased to 0.604, indicating a stronger correlation, this likely reflected cellular uptake saturation rather than true synchronized delivery (Figure S12, Supporting Information). In contrast, the dual‐labeled SQBs, where both fluorophores are physically linked, demonstrated a near 1:1 relationship at 6 h (best‐fit slope 0.90 ± 0.09, R^2^ = 0.78), indicating strong correlation between the two signals from an early stage. This robust correlation was maintained and further strengthened at 24 h, with the slope increasing to 1.06 ± 0.06 and R^2^ value to 0.89, confirming a robust and true synchronized co‐delivery. Additionally, this finding was not specific to the cationic peptide; a similar robust synchronized co‐delivery for the dual‐labeled strategy, in contrast to the stochastic uptake of mixed single‐labels, was also confirmed for SQBs functionalized with the anionic peptide AP2 and the neutral peptide NP5 (Figure 5f,g; Figure S13a–d, Supporting Information). These findings definitively demonstrate that physically linking dual fluorophore reporters on a single DNA origami is a powerful and reliable strategy for ensuring their synchronized delivery to a target cell. It provides a quantitative framework for synchronized co‐delivery that can be applied in future studies using bioactive ligands and functional readouts. This capability is critical for advanced therapeutic strategies, such as synergistic combination therapies, that require the coordinated action of different molecules at the same time and place to achieve their maximum therapeutic effect.

### 2.7 Site‐Specific Hybridization is Required for Biological Function

**Figure 6:**
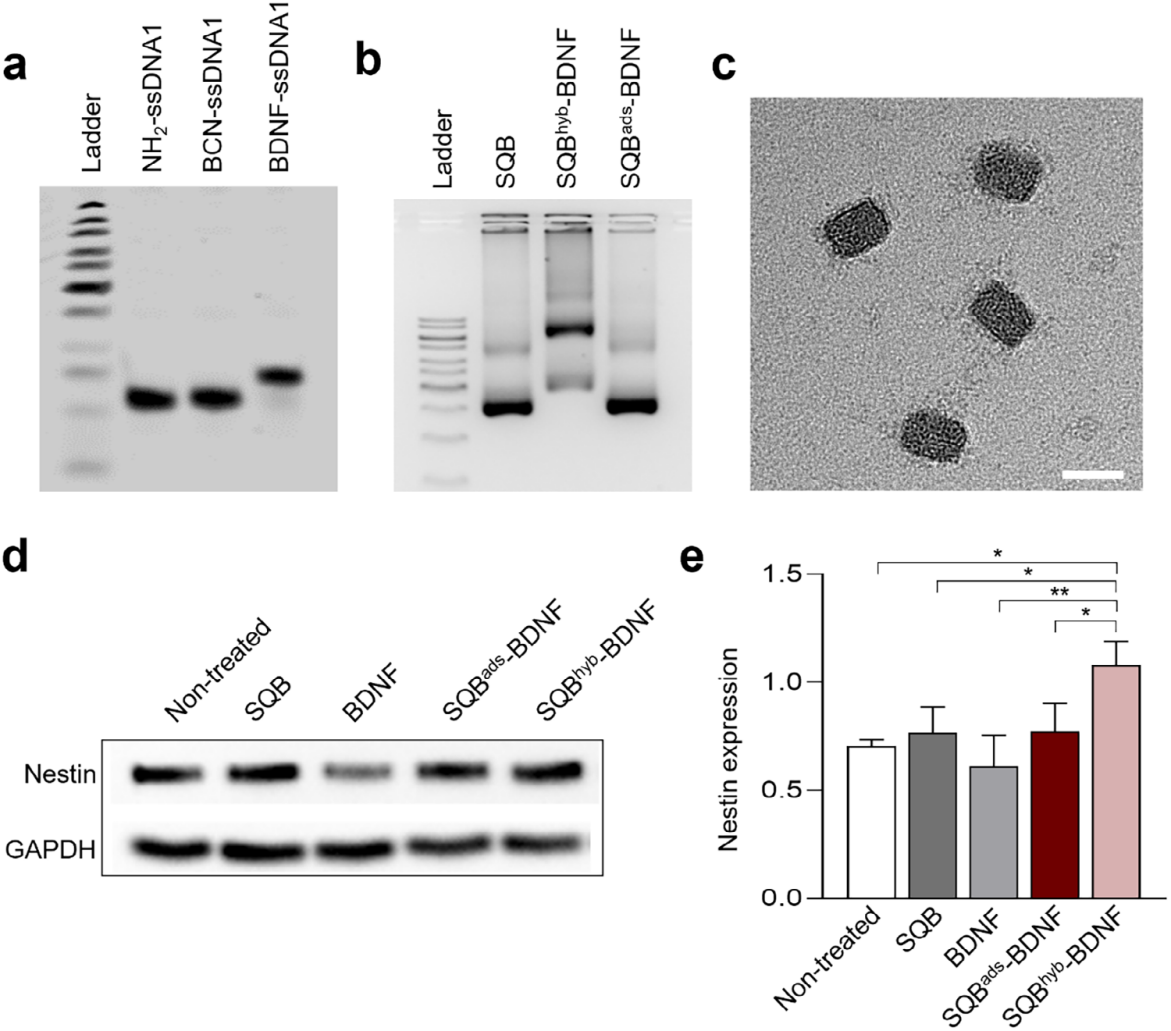
Site‐specific BDNF hybridization on SQBs enhances nestin induction in hMSCs. a) 15% dPAGE confirming the successful synthesis of the BDNF‐ssDNA conjugate (BDNF‐ssDNA1) used for site‐specific hybridization. b) 1.5% agarose gel electrophoresis of the final constructs. Both the site‐specifically hybridized SQB^hyb^‐BDNF and non‐specifically adsorbed SQB^ads^‐BDNF migrate as distinct bands, confirming their structural integrity. c) Representative transmission electron microscopy (TEM) image of the assembled SQB^hyb^‐BDNF showing preservation of the square‐block morphology. Scale bar, 50 nm. d) Representative Western blot of nestin expression (normalized to GAPDH) in hMSCs after 6 days of treatment with the indicated formulations. e) Quantification of nestin expression from (d). The site‐specifically hybridized SQB^hyb^‐BDNF induced a significantly higher nestin expression than all other control groups. Data are presented as mean ± SD (*n* = 3). Statistical analysis was performed by one‐way ANOVA followed by Tukey's test; *p < 0.05, **p < 0.01.

Finally, we sought to determine whether the rigorous control over peptide attachment—achieved through the purification and characterization methods established in this work—is required for the peptide's biological function. To address this, we examined whether the attachment mode of a therapeutic peptide directly influences its biological outcome. We chose a therapeutically relevant brain‐derived neurotrophic factor (BDNF) mimic peptide, which is itself cationic (CP5, net charge +3), making it a perfect model to test the functional consequences of the non‐specific binding. The expression of nestin, a well‐established marker for this differentiation, serves as a clear functional readout of the peptide's bioactivity.^[^ ^14^, ^53^, ^54^, ^55^, ^56^, ^57^ ^]^ To create a direct comparison, we prepared two distinct formulations: SQB^hyb^‐BDNF, prepared via stable, site‐specific DNA hybridization, and SQB^ads^‐BDNF. In SQB^ads^‐BDNF, 37.14 ± 2.26 BDNF peptides per SQB were non‐specifically bound, as confirmed by RP‐HPLC quantification and corroborated by zeta potential measurements (Figures S14a,b and S15, Supporting Information). The successful preparation and structural integrity of both constructs were confirmed by dPAGE, AGE, and TEM analysis, which also verified that a comparable amount of BDNF peptide was initially associated with each formulation (**Figure** **6**a–c). The results from Western blot analysis revealed a significant difference in the biological response (Figure 6d,e; Figure S16, Supporting Information). While the non‐specifically adsorbed peptide (SQB^ads^‐BDNF) elicited only a modest increase in nestin expression, the site‐specifically hybridized SQB^hyb^‐BDNF induced a significantly more potent biological response.^[^ ^58^, ^59^ ^]^

The superior biological function of SQB^hyb^‐BDNF stems directly from the stability of the peptide‐nanostructure linkage. Non‐specific electrostatic adsorption is a weak and reversible interaction that is likely disrupted in complex biological environment, causing the peptide cargo to prematurely dissociate from the nanostructure and resulting in a low effective dose. In contrast, site‐specific DNA hybridization provides a robust, covalent‐like anchor that secures the peptide payload. This stable attachment ensures the effective presentation of the peptide to the cells, leading to a more potent biological response.

Furthermore, this stable and site‐specific attachment is a fundamental prerequisite for leveraging the core advantage of DNA origami: the precise nanoscale arrangement of ligands. It has been increasingly demonstrated that the biological response to a nanostructure is not only determined by the presence of a ligand, but by its precise valency and spatial organization. For example, the exact nanoscale spacing and number of insulin molecules on a DNA nanorod have been shown to dictate the strength of insulin receptor activation, while the valency of viral antigens displayed on an origami scaffold can determine the potency of B cell.^[^ ^5^, ^6^ ^]^ Crucially, as we have shown in a previous study, tuning the spacing of CpG adjuvants to an optimal distance of 3.5 nm on a DNA origami vaccine is critical for maximizing the Th1‐polarized immune responses required for effective cancer therapy.^[^ ^3^ ^]^ Recent studies have further expanded this concept by demonstrating how programmable multivalency on DNA origami can regulate diverse biological interactions—from antigen presentation and receptor clustering to immune activation—highlighting the generality and translational potential of nanoscale ligand patterning.^[^ ^7^, ^8^, ^9^ ^]^ Such precise spatial control is only achievable through robust, site‐specific conjugation and would be impossible with the uncontrolled, random loading of electrostatic adsorption.

These findings provide definitive functional evidence that a stable, site‐specific linkage is superior to simple adsorption for this therapeutic application. The data confirms that the precise, site‐specific conjugation and purification methods established in this work are a fundamental requirement for creating DNA nanodevices with robust and predictable biological activity.

## 3 Conclusion

In this work, we addressed a critical and previously overlooked challenge in the functionalization of DNA origami: the non‐specific electrostatic binding of cationic peptides to the anionic DNA framework. We showed quantitatively that this leads to uncontrolled and excessive peptide loading, far exceeding the intended number of binding sites. Our analysis also revealed that this aggregation behavior, while driven by electrostatics, is highly peptide‐specific and not a universal trait of all cationic molecules.

To mitigate this issue, we established a practical purification strategy using optimized PEG precipitation. Our findings provide a clear design guideline: the number of purification cycles must be tailored to the peptide's charge, with cationic peptides requiring extensive treatment (≥7 cycles) while anionic peptides need only minimal purification (1–2 cycles) to achieve precise stoichiometric loading.

Finally, we provided functional evidence that this rigorous control is essential for biological activity. We demonstrated that a therapeutic BDNF peptide is potent only when attached via a stable, site‐specific bond, as non‐specifically adsorbed peptides are largely inactive. Furthermore, we showed that only these precisely defined nanostructures can ensure the synchronized co‐delivery of multiple ligands to the same cell. The methods and principles established in this work provide a set of critical guidelines necessary for the rational design of reliable and functionally predictable DNA nanodevices.

## 4 Experimental Section

### Material Sources

ssDNA staples and anti‐handle ssDNA strands were synthesized by IDT (IA, US). All peptides were synthesized by Beadtech (Incheon, Korea). (1R,8S,9s)‐Bicyclo[6.1.0]non‐4yn‐9‐ylmethyl N‐succinimidyl carbonate (BCN‐NHS, #1426827‐79‐3), ethyl alcohol (ethanol, #459834), sodium chloride (NaCl, #S9888), magnesium chloride hexahydrate (MgCl_2_, #M2670), dimethyl sulfoxide (DMSO, #276855), and polyethylene glycol‐8000 (PEG‐8000, #89510) were purchased from Sigma Aldrich (MO, US). 10× Tris‐EDTA (TE) was purchased from HanLAB (Gyeonggi‐do, Korea) while DNase I was purchased from New England Biolabs (MA, US). SYBR Gold Nucleic Acid Gel Stain (#S11494) was supplied by Thermo Fisher Scientific (MA, US). Methoxy‐PEG‐block‐poly(L‐lysine hydrochloride) (K_10_‐PEG_5k_) was purchased from Alamanda Polymers (AL, US). TEM grids (CF200‐CU) were purchased from Electron Microscopy Sciences (PA, US). Human mesenchymal stem cells (hMSC, PT‐2501) and Mesenchymal Stem Cell Growth Medium Bullet Kit (MSCGM, PT‐3001) were obtained from Lonza (Basel, CH). Rabbit anti‐human nestin antibody (#ab105389) and mouse anti‐human GAPDH antibody (#ab8245) were purchased from Abcam (Cambridge, UK). Goat anti‐rabbit HRP‐conjugated antibody (#1705046) and goat anti‐mouse HRP‐conjugated antibody (#1705047) were purchased from Bio‐Rad (CA, US).

### Preparation and Characterization of Peptide‐ssDNA or Fluorescent Dye‐ssDNA Conjugates

Anti‐handle ssDNA strands used were ssDNA1 (5′‐GCTGTTAGAGAATGAGAGTCG‐3′), ssDNA2 (5′‐AGTGATGTGAGACCATGTGAG‐3′) and ssDNA3 (5′‐TTCTAGGGTTAAAAGGGGACG‐3′) (Table S1, Supporting Information). For peptide conjugation, 5′ amine modified ssDNA was first converted to BCN‐ssDNA by reacting with BCN‐NHS ester at a molar ratio 1:10 in a 1:1 mixture of 1× TE buffer (10 mM Tris‐HCl, 1 mm EDTA, pH 8.0) and DMSO and incubated overnight at 25 °C with shaking. To remove excess BCN‐NHS, resulting BCN‐ssDNA was precipitated by 2.5 volumes of ethanol and 0.1 volumes of 3 m sodium acetate (pH 5.2), followed by incubation at −20 °C for 1 h. Pellets were collected by centrifugation at 16 000 × g for 20 min at 4 °C, washed once with 75% ethanol under the same conditions, air‐dried, and resuspended in 1× TE buffer. BCN‐ssDNA was then reacted with azide‐functionalized peptides at a 1:10 ssDNA‐to‐peptide molar ratio in 1× TE buffer, with 7% DMSO added when required for solubility, and incubated overnight under the same conditions. For several peptides with poor solubility, the reactions were also carried out in buffer conditions containing 90% DMSO. Removal of excess peptides was carried out by ethanol precipitation, and the final pellets were resuspended in 1× TE buffer. For fluorescent dye conjugation, 5′ amine modified ssDNA1 or ssDNA2 was reacted with either Cyanine 5.5 NHS ester or fluorescein isothiocyanate (FITC) at a 1:10 molar ratio, in a 1:1 mixture of 1× TE buffer and DMSO, and incubated overnight at 25 °C with shaking. Excess dye was removed by ethanol precipitation as above. Conjugation yield and product purity were evaluated by gel electrophoresis and ImageJ as described below.

### Turbidity Assay

ssDNA stock solutions were prepared in 1× TE buffer to a final concentration of 0.07 mm. For salt‐dependent measurements, the buffer was first adjusted with a concentrated NaCl solution to achieve the target NaCl concentration, and the ssDNA was allowed to equilibrate. To initiate the assay, peptide solutions were added to the ssDNA‐containing buffer at a tenfold molar excess. Turbidity was monitored immediately by recording the optical density at 350 nm (OD_350_) in a 96‐well plate using a BioTek Synergy H1 microplate reader, with blank‐subtracted values reported.

### Centrifugation‐Based Solubility Assay

To determine the soluble fraction of ssDNA after mixing with peptides, the mixtures were prepared as described above and then centrifuged at 16 000 × g for 5 min at room temperature. The supernatants were carefully collected, and the ssDNA concentration was measured by absorbance at 260 nm (OD_260_) on a NanoDrop spectrophotometer. The soluble fraction was calculated as OD_260,supernatant_/OD_260,initial_, where OD_260,initial_ corresponds to the absorbance of the ssDNA reference solution (0.07 mm in 1× TE buffer without peptide or salt).

### Quantification of Conjugation Yield

The yield of the peptide‐ssDNA conjugation reaction was assessed from dPAGE gel images using ImageJ. Rectangular regions of interest (ROIs) were drawn around the bands corresponding to the final conjugate and the unreacted ssDNA. After background subtraction, the integrated density of each band was measured. The yield (%) was calculated using the following equation:

(1) $$\mathrm{Yield}\;(\%)=\frac{I_{conjugate}}{I_{conjugate}+I_{unreacted}}\times 100$$

where I_conjugate_ and I_unreacted_ represent the integrated band densities of the conjugated product and the unreacted ssDNA, respectively.

### DNA Origami SQB Folding and Purification

The p8634 scaffold strand was produced from M13 phage replication in *Escherichia coli*, following previously reported methods (Table S2, Supporting Information). For SQB assembly, the scaffold was combined with a fivefold molar excess of staple strands in folding buffer (1× TE with 12 mm MgCl_2_) containing handle sequences for subsequent peptide or dye conjugation. The handle sequences used for each purpose are provided in Tables S3–S9 (Supporting Information). The mixture was first heated to 80 °C for 15 min to denature the strands, and then annealed by gradually cooling from 50 to 40 °C at a rate of 0.1 °C every 10 min and 48 s. Excess staples were removed by PEG precipitation, and successful SQB formation was validated by agarose gel electrophoresis (1.5%) and TEM imaging.

### PEG Precipitation of SQBs and Ligand‐Conjugated SQBs

SQBs or SQB‐ligand conjugates were purified by PEG precipitation using 22% (w/v) PEG‐8000 and 510 mm NaCl. For single‐step purification of samples in 12 mm MgCl_2_ TE buffer, mixing 1:1 (v/v) with PEG solution (8 mM MgCl_2_) yielded a final MgCl_2_ concentration of 10 mm. For repeated precipitation of SQB‐ligand conjugates already suspended in 10 mm MgCl_2_ TE buffer, the same 1:1 mixing with PEG solution containing 10 mm MgCl_2_ ensured that the final concentration remained 10 mm. Pellets were collected by centrifugation and resuspended in 1× TE buffer with 10 mm MgCl_2_. Concentrations were determined using a NanoDrop spectrophotometer.

### Conjugation of Peptide‐ssDNA or Fluorescent Dye‐ssDNA Conjugates onto SQB and Purification

SQBs were incubated with peptide‐ssDNA or fluorescent dye‐ssDNA conjugates at a fivefold molar excess relative to the number of available handle sites. The available handle site corresponds to the sequence complementary to the anti‐handle ssDNA used for peptide‐ssDNA or fluorescent dye‐ssDNA conjugation. The mixture was maintained at 37 °C with shaking at 800 rpm for 2 h to facilitate annealing of the peptide‐ssDNA or fluorescent dye‐ssDNA conjugates onto the SQBs. Excess, unbound peptide‐ssDNA or fluorescent dye‐ssDNA conjugates were subsequently removed by PEG precipitation, and successful formation of SQBs conjugated with peptides or fluorescent dyes was verified by AGE and HPLC analysis.

### Gel Electrophoresis

The synthesis and purity of BCN‐ssDNA conjugates and peptide‐ssDNA or fluorescent dye‐ssDNA conjugates were examined by dPAGE. Samples were resolved on 15% polyacrylamide gels in 0.5× TBE buffer at 200 V, stained with SYBR Gold, and visualized using an iBright imaging system. SQBs and ligand‐conjugated SQBs were characterized by agarose gel electrophoresis using 1.5% agarose gels prepared in 0.5× TBE buffer run at 70 V for 2 h.

### Transmission Electron Microscopy (TEM)

The morphology and size of SQBs and peptide‐conjugated SQBs were examined by negative‐stain TEM using a Tecnai microscope. Samples were diluted to 8–10 nm in 1× TE buffer with 10 mm MgCl_2_, and 4 µL was applied to a TEM grid for 4 min. Excess liquid was removed, followed by the addition of 4 µL of 2% uranyl acetate for 1 min. The stain was blotted off, and grids were dried overnight before imaging.

### DNase I Digestion and HPLC Analysis

For HPLC‐based quantification of SQB–bound peptides, samples were digested with DNase I. SQBs were incubated with 0.05 U/µg DNase I (New England Biolabs, M0303S) in 1× DNase I buffer (prepared from 10× stock diluted in water, Gibco) at 37 °C for 2 h. Quantification of peptides was performed using a gradient RP–HPLC method. Analyses were carried out on a ZORBAX StableBond C18 column (80 Å, 5 µm, 4.6 × 150 mm; Agilent) fitted to an Agilent 1260 Infinity II system. The mobile phase A was 0.1% formic acid in HPLC–grade water, and mobile phase B was 0.1% formic acid in acetonitrile. A linear gradient from 95:5 to 5:95 (A/B, v/v) over 20 min was applied, followed by 2 min at 5:95 and re‐equilibration at 95:5. The total run time was 25 min for each 60 µL injection. The flow rate was 1 mL min^−1^, and UV detection was set at 220 nm. The retention times for CP4 and AP2 were approximately 6.8 and 10.0 min, respectively. Linear calibration curves for CP4 and AP2 were obtained in the ranges of 2.84–45.4 µg mL^−1^ (R^2^ > 0.999) and 1.73–55.3 µg mL^−1^ (R^2^ > 0.996), respectively (*n* = 3).

### K_10_‐PEG_5k_ Coating of SQBs

Prior to in vitro studies, SQBs either unmodified or conjugated with peptides and fluorophores, were coated with K_10_‐PEG_5k_ to improve protection against enzymatic degradation. For coating, K_10_‐PEG_5k_ and SQBs were combined at an N:P ratio of 1:1 (nitrogen atoms in amines relative to phosphate groups in DNA), and the mixture was incubated for 1 h at RT.

### Cellular Uptake of Peptide‐Functionalized SQBs

hMSCs were seeded on poly‐D‐lysine–coated coverslips in 12‐well plates at 5 × 10^3^ cells/cm^2^ and cultured overnight. Cells were incubated with 10 nm SQBs functionalized with cationic (+3), neutral (0), or anionic (–3) peptides. For this study, the opposite face of each SQB was labeled with FITC. After treatment, cells were rinsed with PBS, fixed with 4% paraformaldehyde for 20 min at room temperature, washed, and stained with Hoechst 33342. Coverslips were mounted with antifade medium and dried overnight before imaging on a TCS SP8 confocal microscope (Leica Microsystems).

### Quantification of Cellular Uptake Analysis by Mean Fluorescence Intensity (MFI)

Confocal images were randomly acquired from treated cells to assess overall intracellular uptake. The total FITC fluorescence intensity within each image was measured using ImageJ, and DAPI‐stained nuclei were counted to determine the number of cells per image. The total FITC intensity of each image was then divided by the number of nuclei to calculate the MFI per cell, providing a measure of intracellular uptake of peptide‐functionalized SQB.

### Single‐Cell Quantification of Co‐Delivery

Single‐cell quantification was performed to evaluate co‐delivery using ImageJ. FITC, Cy5.5, and DIC channels were imported into ImageJ and converted to 16‐bit grayscale. Cell boundaries were traced on DIC images to define ROIs, which were applied to fluorescence channels. After thresholding and binarization, spots were segmented and quantified using the “Analyze Particles” function, which also resolved closely spaced or partially overlapping puncta. Puncta were identified as fluorescent‐positive regions rather than by intensity values. The number of FITC‐ and Cy5.5‐positive spots per cell was exported and visualized as scatter plots in GraphPad Prism, with FITC counts on the x‐axis and Cy5.5 counts on the y‐axis.

### Western Blotting

For nestin expression analysis, hMSCs were seeded in 6‐well plates at a density of 5 × 10^3^ cells/cm^2^ and incubated overnight. Treatments were performed three times, with medium changed every other day. On day 6, the treated hMSCs were harvested and lysed in radioimmunoprecipitation assay (RIPA) buffer supplemented with a protease inhibitor cocktail. Protein concentrations in the lysates were quantified using a bicinchoninic acid (BCA) assay. Equal amounts of protein were separated on 8% SDS‐PAGE gels and transferred to polyvinylidene difluoride (PVDF) membranes. Membranes were blocked with 5% skim milk for 1.5 h at RT and then incubated overnight at 4 °C with primary antibodies. The primary antibodies used were rabbit anti‐human nestin (1:500) and mouse anti‐human GAPDH (1:1000). After washing, the membranes were incubated with HRP‐conjugated goat anti‐rabbit or goat anti‐mouse secondary antibodies for 1 h at RT. Protein bands were visualized using enhanced chemiluminescence (iBright, Thermo Fisher) and quantified with ImageJ, with expression levels normalized to GAPDH.

### Statistical Analysis

All quantitative results were processed using GraphPad Prism software and are expressed as mean ± standard deviation (SD). For comparisons among multiple groups, either one‐way ANOVA was performed, followed by Tukey's or Sidak's post hoc multiple‐comparison tests, as indicated in the respective figure legends. For two‐group comparisons, unpaired two‐tailed *t*‐tests were used. Statistical significance was denoted as *p < 0.05, **p < 0.01, ***p < 0.001, and ****p < 0.0001.

## Conflict of Interest

The authors declare no conflict of interest.

## Supporting information



Supporting Information

## Data Availability

The data that support the findings of this study are available from the corresponding author upon reasonable request.
