# Supplementary material for: Characterization of Non‐Specific Electrostatic Interactions of Cationic Peptides with DNA Origami and Their Functional Consequences
Source: Small Methods. 2025 Dec 5;10(1):e01936. doi: 10.1002/smtd.202501936 (PMC12790360; doi:10.1002/smtd.202501936)
Supplement: Supplementary file 1 — Supporting Information [file SMTD-10-e01936-s001.docx]

**Supporting Information**

**Characterization of Non-Specific Electrostatic Interactions of Cationic Peptides with DNA Origami and Their Functional Consequences**

Seung Hyun Kang, Oheun Kwon, Bo Kyung Cho, Seungmin Yoo, Jin Myeong Wang, Youngjin Choi, Hong Yeol Yoon*, Jungkyu Choi*, Ju Hee Ryu*

S. H. Kang, O. Kwon, B.K. Cho, S. Yoo, J.M. Wang, Y. Choi, H. Y. Yoon, J. H. Ryu

Medicinal Materials Research Center, Biomedical Research Institute, Korea Institute of Science and Technology (KIST), Seoul 02792, Republic of Korea
E-mail: seerou@kist.re.kr

S. H. Kang, J. Choi

Department of Chemical and Biological Engineering, Korea University, Seoul 02481, Republic of Korea

E-mail: jungkyu_choi@korea.ac.kr

O. Kwon, S. Yoo, J. H. Ryu

KU-KIST Graduate School of Converging Science and Technology, Korea University, Seoul 02841, Republic of Korea

E-mail: jhryu@kist.re.kr

Keywords: DNA origami, cationic peptide, non-specific binding, stoichiometric control, electrostatic interaction

**Methods**

*Dynamic Light Scattering (DLS) Measurements*: Measurements were performed using a Zetasizer Nano ZS (Malvern Panalytical). Samples (18 nM SQBs in 1× TE buffer with 10 mM MgCl₂) were analyzed at 25 °C. For size measurements, samples were loaded into disposable solvent-resistant micro cuvettes (ZEN0040). A backscatter angle of 173° was used, with a 60 s equilibration before measurement. Three technical replicates were collected per sample. Data were analyzed with Zetasizer software v7.13, applying the default water refractive index and viscosity settings.

**
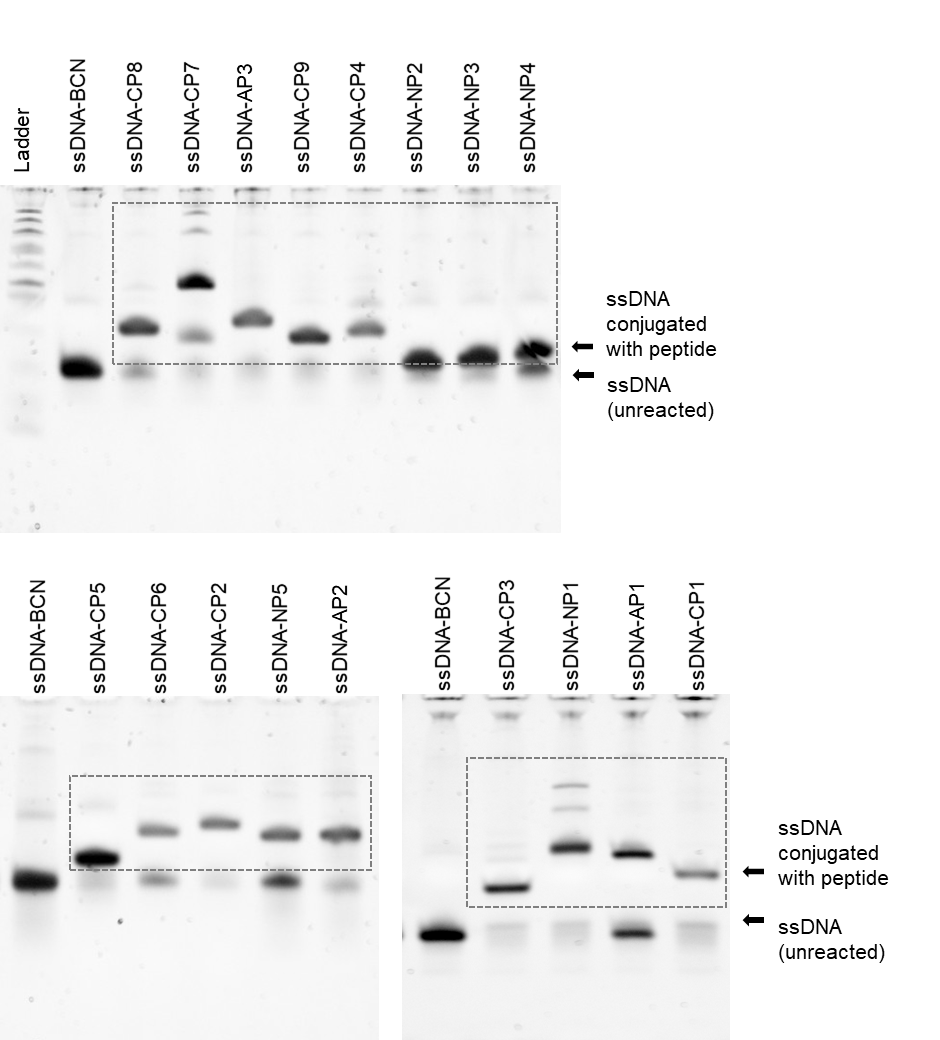
**

**Figure S1. Confirmation of peptide–ssDNA conjugation by denaturing polyacrylamide gel electrophoresis (dPAGE).** 15% dPAGE gels showing mobility shifts of ssDNA after conjugation with the peptide panel (CP, AP, NP series). Each conjugated product migrates more slowly than unreacted BCN-ssDNA, consistent with increased molecular weight. Multiple bands were observed for several conjugates, which likely reflect diastereomers and minor reaction variants of the SPAAC product. All conjugate bands were collectively quantified as product, with the unreacted ssDNA band excluded. The corresponding well regions have been included to visualize any immobile, aggregated fraction of the peptide–DNA conjugates. While minor aggregation was observed in the well for the highly cationic CP1 conjugate, no significant aggregation was seen for the other conjugates. This is expected because the conjugation products were analyzed under dPAGE conditions, including a heating step that dissociates electrostatically-driven aggregates, allowing all species to migrate into the gel matrix. Conjugation yields were quantified from band intensities (summarized in Table 1).

**
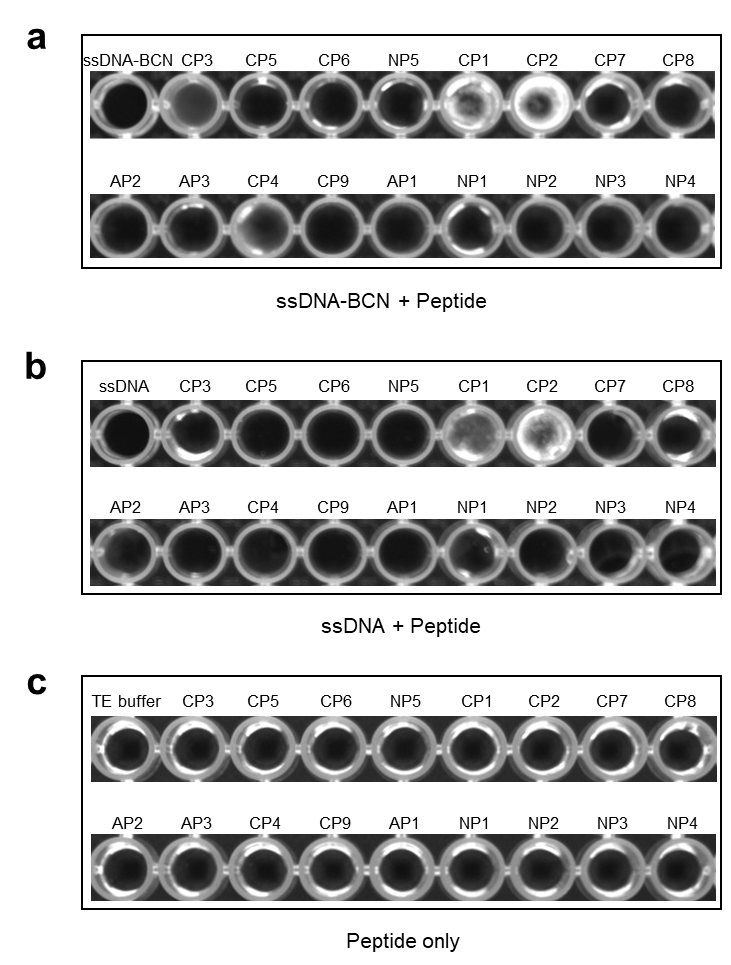
**

**Figure S2. Turbidity formation of peptide-ssDNA mixtures.** Representative images of 96-well plate assays showing turbidity upon immediately mixing peptides with (a) BCN-modified ssDNA (b) unmodified ssDNA (c) Peptides alone (without ssDNA) under identical assay conditions, showing no visible turbidity.


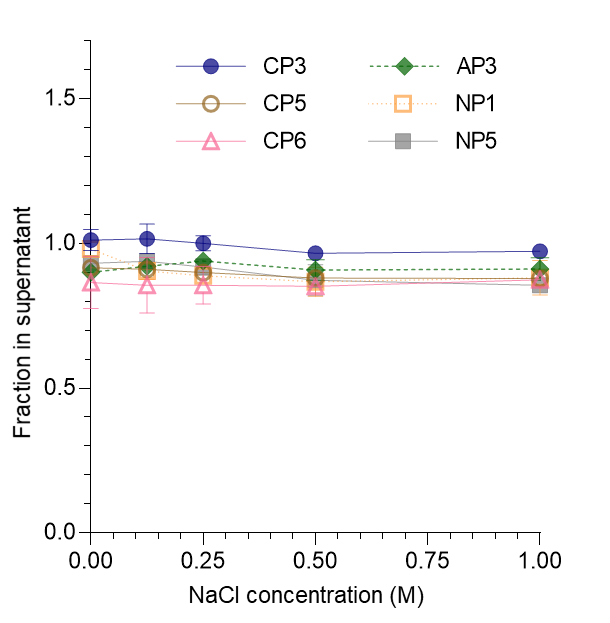


**Figure S3. Pull-down assays for additional representative peptide–ssDNA pairs.** In addition to the four peptides shown in Figure 1f, pull-down analysis was performed on six additional peptides—CP3 (+2), CP5 (+3), CP6 (+2), AP3 (−1), NP1(0) and NP5 (0)—selected to cover distinct charge and various properties. ssDNA depletion were observed, consistent with their weaker aggregation tendencies compared with CP1 and CP2. These data expand the experimental coverage across peptide properties and confirm the overall trend that aggregation does not directly correlate with conjugation yield.


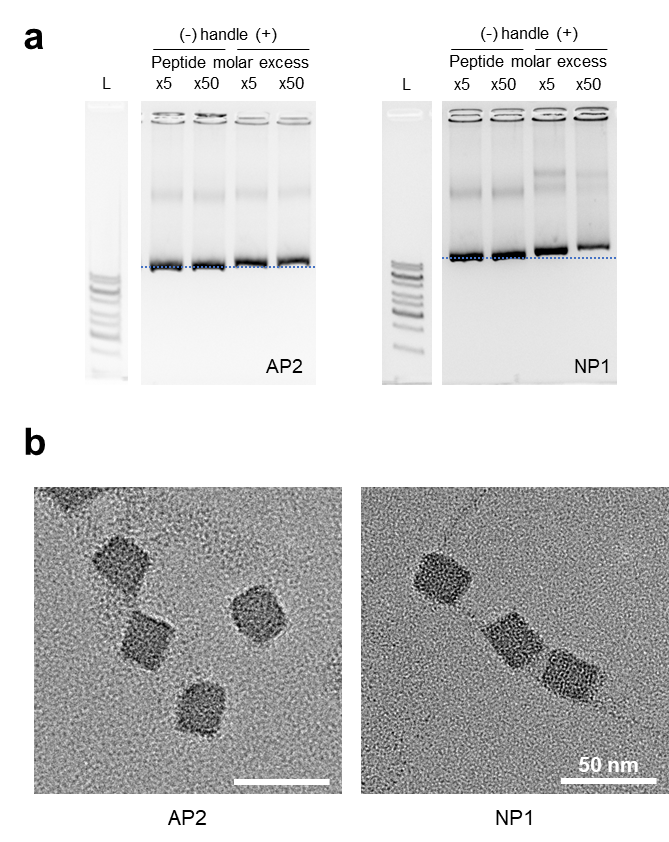


**Figure S4. Validation of peptide-conjugated SQBs via AGE and TEM** (a) AGE data of SQB incubated with ssDNA–AP2 (anionic) and ssDNA–NP1 (neutral) conjugates under the indicated peptide molar excess (×5, ×50). Lanes are shown after PEG precipitation. Handle (–) indicates samples incubated with peptide lacking the anti-handle, whereas handle (+) indicates samples incubated with ssDNA-peptide bearing the anti-handle that can be annealed to SQB handles. Dotted lines have been added to guide the eye and highlight the band shift of the handle (+) lanes relative to the handle (-) lane. (b) TEM images of AP2- and NP1-conjugated SQBs show preservation of the intended square-block architecture without discernible deformation or aggregation. The scale bar indicates 50 nm.


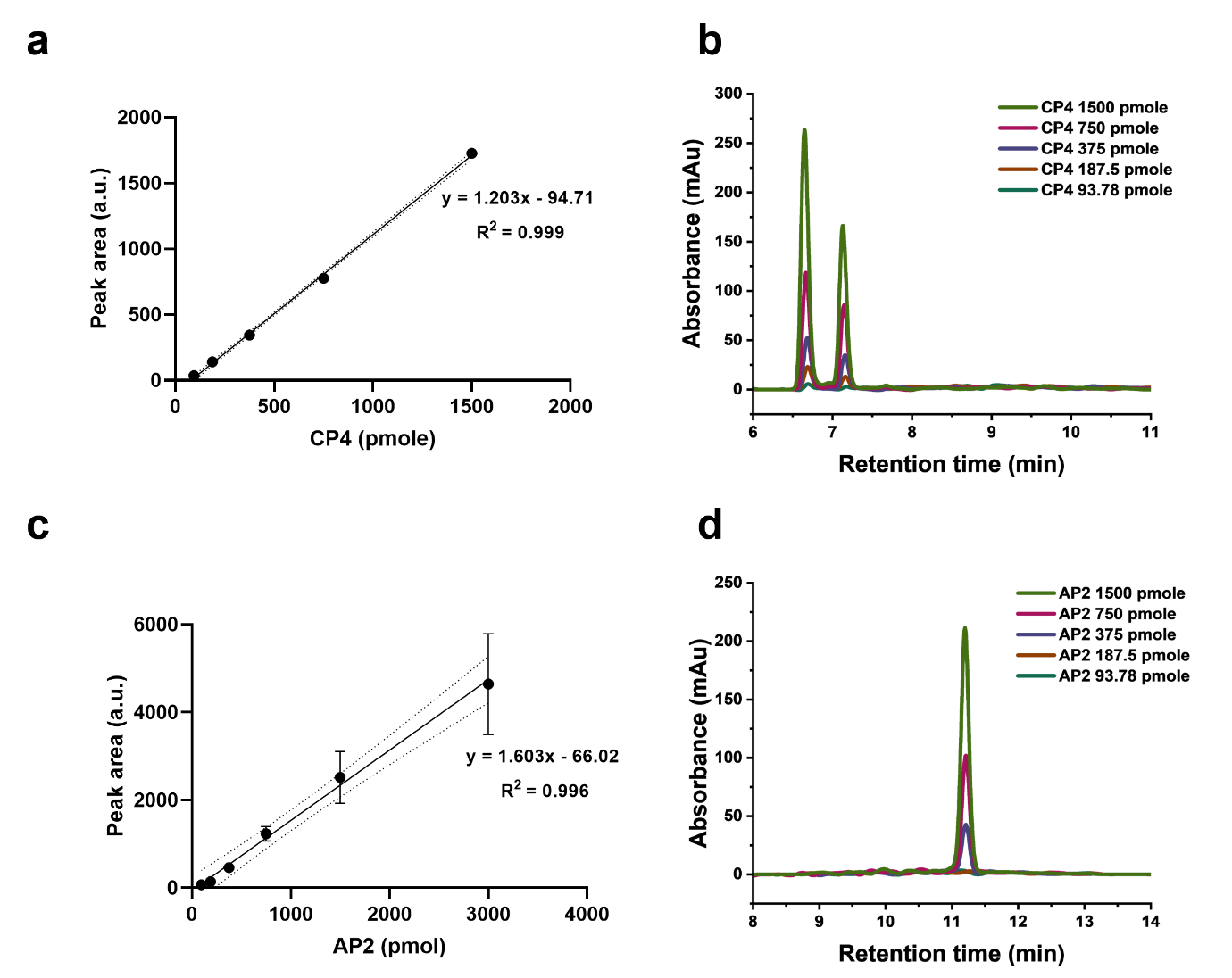


**Figure S5. Standard calibration curves and chromatographic identification of intact CP4 and AP2 peptides by RP-HPLC.** (a) Standard calibration curve of the CP4 peptide obtained by RP-HPLC. Linear regression yield y = 1.203x – 94.71 (R2 = 0.999). (b) Overlaid chromatograms of the CP4 peptide standard. Multiple peaks were consistently observed in both CP4 peptide standard and the sample. Among them, the earliest-eluting peak (6.8-7.1 min) consistently exhibited the highest intensity and reproducibility across replicate injections, indicating that it represents the predominant intact peptide species under the applied chromatographic conditions. This peak was therefore considered to represent the intact peptide species and was selected as the reference peak for quantitative analysis. (c) Standard calibration curve of the AP2 peptide obtained by RP-HPLC. Linear regression yielded y = 1.603x – 66.02 (R2 = 0.996). (d) Overlaid chromatograms of the AP2 peptide standard. The AP2 peptide standard exhibited a single dominant peak, while multiple peaks were observed in the sample. Among these, the peak eluting at 9.5-11 min matched the retention time of the standard and was reproducibly observed across replicate injections. This peak was therefore considered to represent the intact peptide species and was selected as the reference peak for quantitative analysis.

**
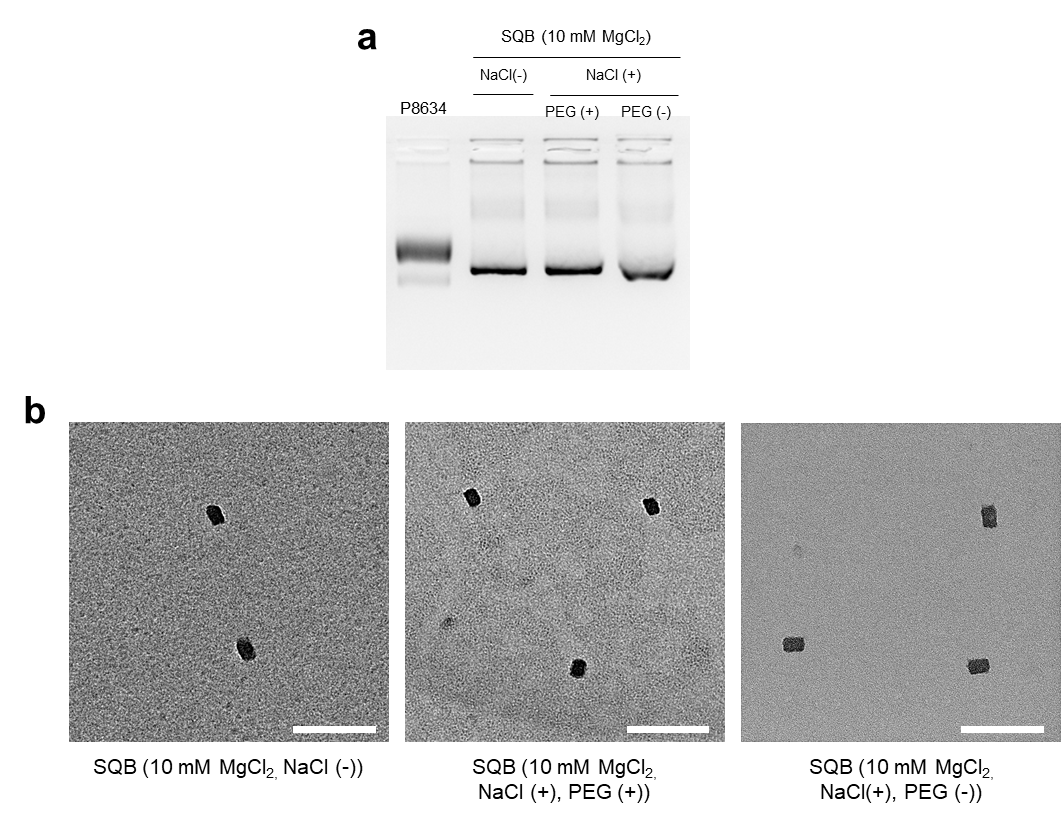
**

**Figure S6. Stability of SQB DNA origami in high-salt buffers.** (a) AGE data of SQB assembled in 10 mM MgCl₂ and incubated with or without high NaCl, with PEG precipitation indicated as (+/−). The intact, well-defined bands indicate that elevated NaCl did not compromise SQB structural integrity. (b) TEM images of SQB under the same buffer conditions, showing preserved morphology without apparent aggregation. Scale bars are 100 nm.


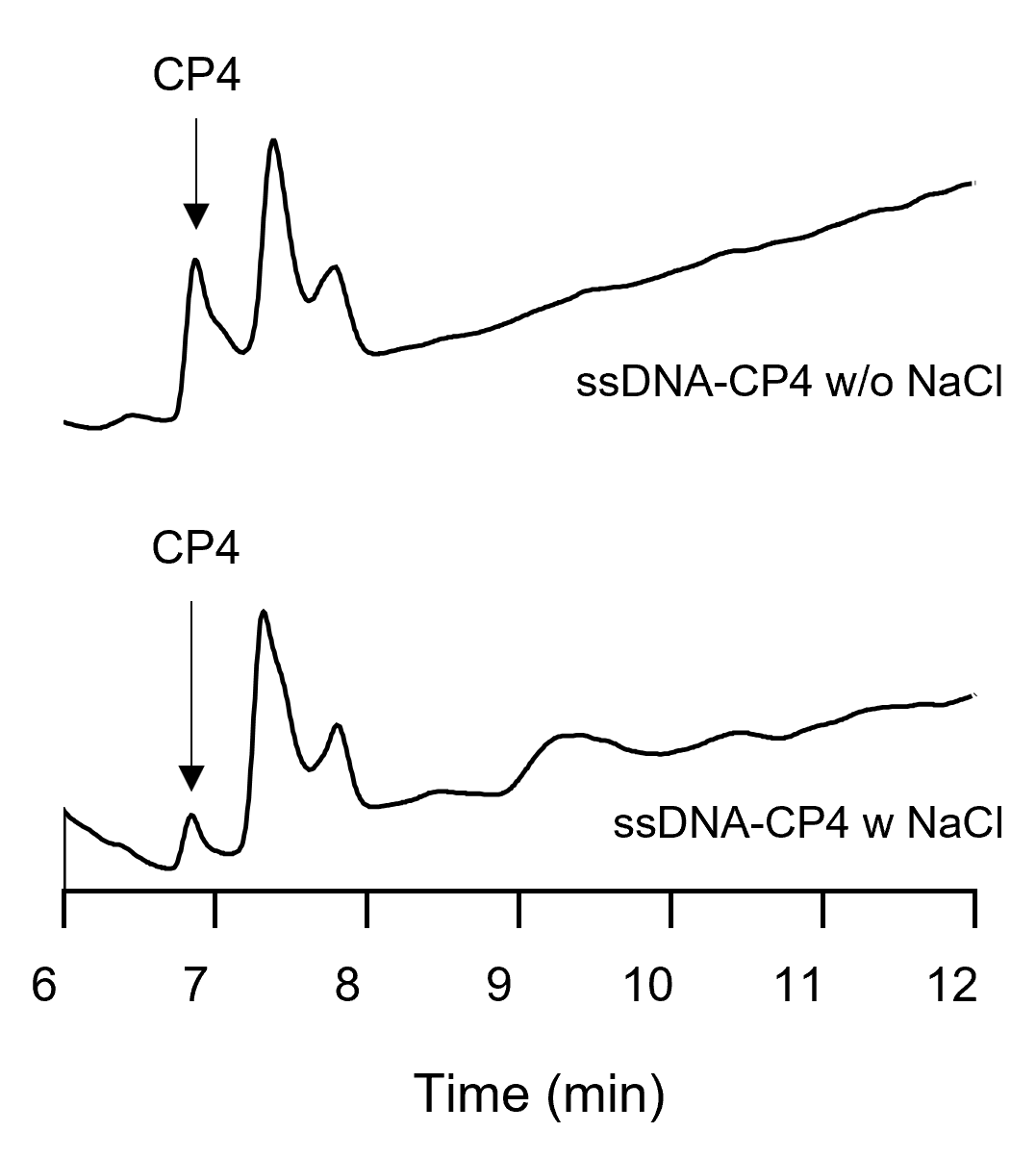


**Figure S7. CP4 HPLC Chromatograms acquired after incubation in the presence or absence of NaCl (Quantification: 6.8-7.1 min)** Representative HPLC chromatograms of CP4-functionalized samples acquired with and without added NaCl during incubation.


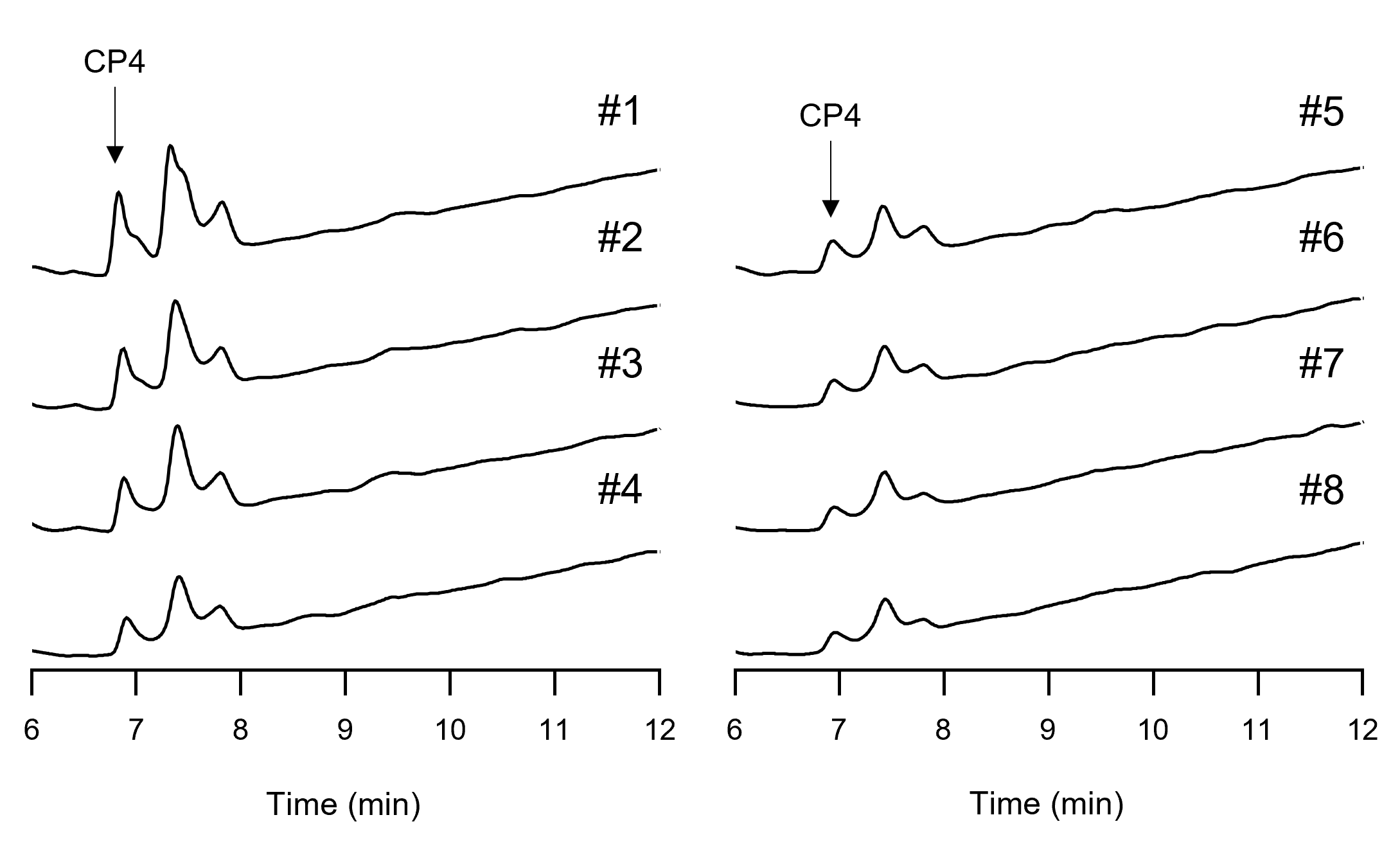


**Figure S8. CP4 HPLC Chromatograms over PEG Precipitation Cycles 1–8 (Quantification: 6.8-7.1 min)** Representative HPLC chromatograms for CP4 after cycles #1–#8. The peak area at 6.8-7.1 min is used for CP4 quantification.


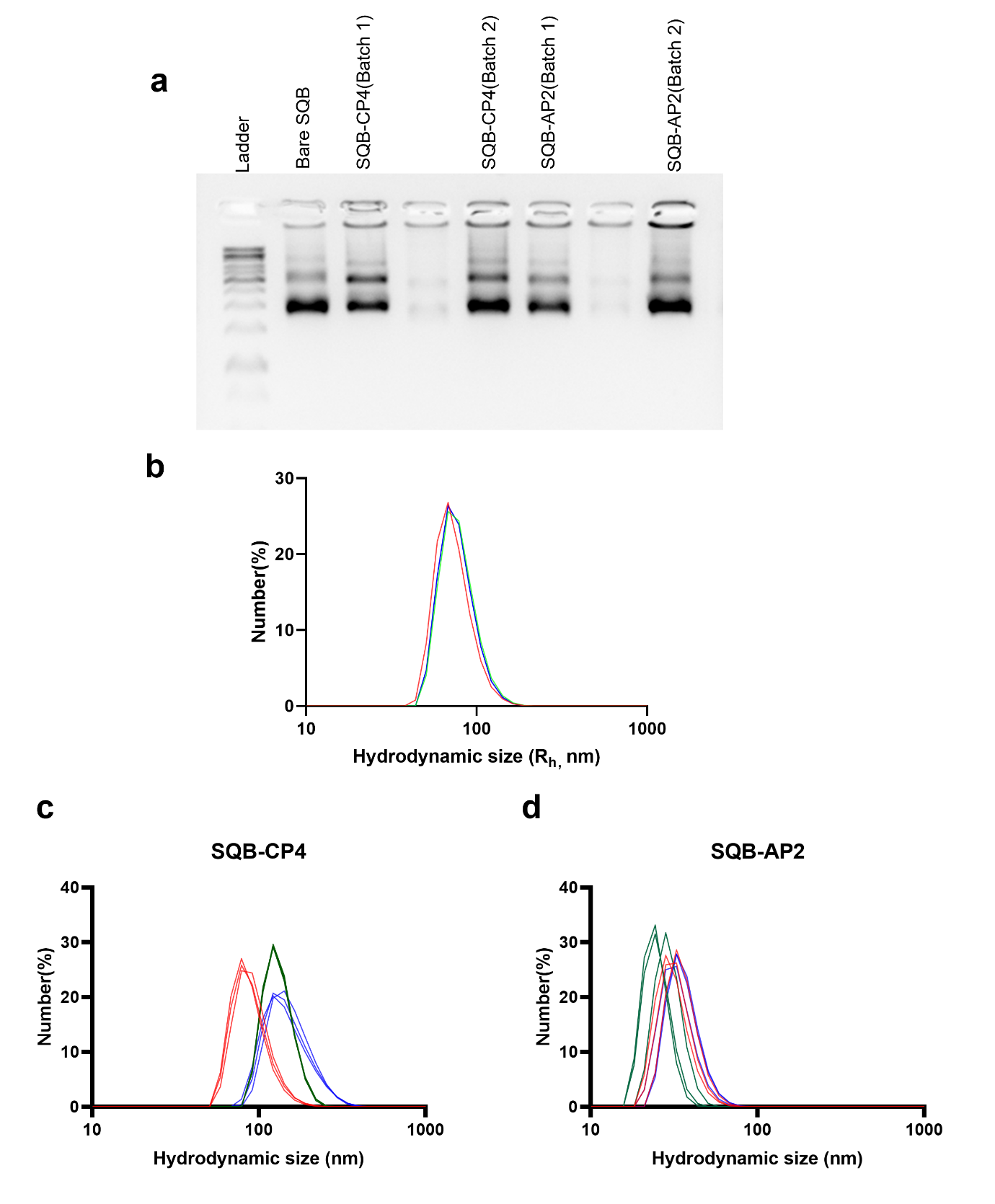


**Figure S9.** (a) Agarose gel electrophoresis image of SQB DNA origami, conjugated with CP4 and AP2 of a distinct batch. (b-d) Hydrodynamic size distribution of SQB DNA origami conjugated with (b) no peptide, (c) CP4, and (d) AP2.

Each color represents one of the three distinct batches, while the line represents three different technical replicates for each batch. The hydrodynamic size was measured using Dynamic Light Scattering (DLS).


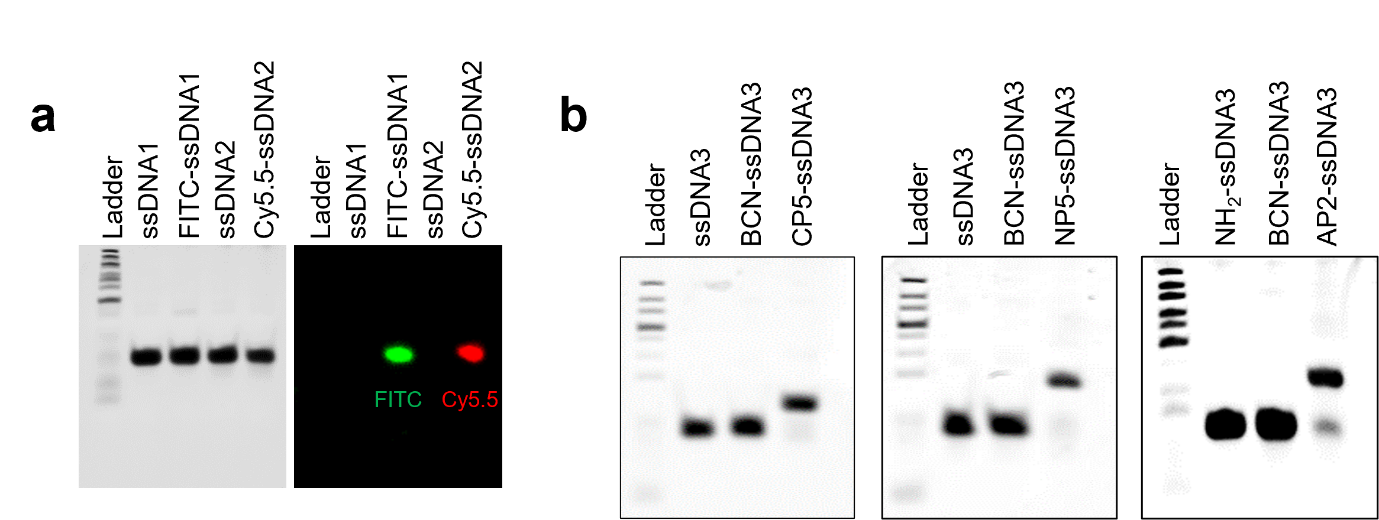


**Figure S10. Gel-based validation of fluorescent dye-ssDNA or peptide-ssDNA conjugates and their incorporation into SQBs.** (a) 15% dPAGE of fluorescent dye-ssDNA conjugates (FITC-ssDNA1 and Cy5.5-ssDNA2); left, nucleic acid channel; right, in-gel fluorescence acquired in FITC and Cy5.5 channels, confirming successful dye conjugation and clean products. (b) 15% dPAGE of peptide-ssDNA conjugates (CP5-ssDNA3, NP5-ssDNA3, and AP2-ssDNA3), verifying successful conjugation.


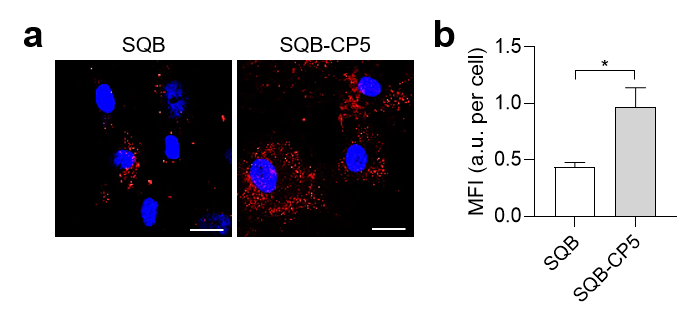


**Figure S11. Comparison of cellular uptake between peptide-free SQBs and cationic peptide-functionalized SQBs.** (a) Representative confocal images of human mesenchymal stem cells (hMSCs) after 6 h incubation with Cy5.5-labeled SQBs or Cy5.5-labeled SQB-CP5. SQBs were visualized by Cy5.5 fluorescence (red), and nuclei were stained with DAPI (blue). A modest increase in intracellular fluorescence was observed for SQB-CP5 compared with peptide-free SQBs, indicating enhanced cellular uptake. Scale bars, 20 µm. (b) Corresponding quantification of Mean Fluorescence Intensity (MFI) per cell. MFI was calculated by normalizing the total cytoplasmic Cy5.5 intensity to the number of nuclei. Data are presented as mean ± SD (n=3 randomly selected images per group). Statistical significance was analyzed by unpaired two-tailed t-test, *p < 0.05.


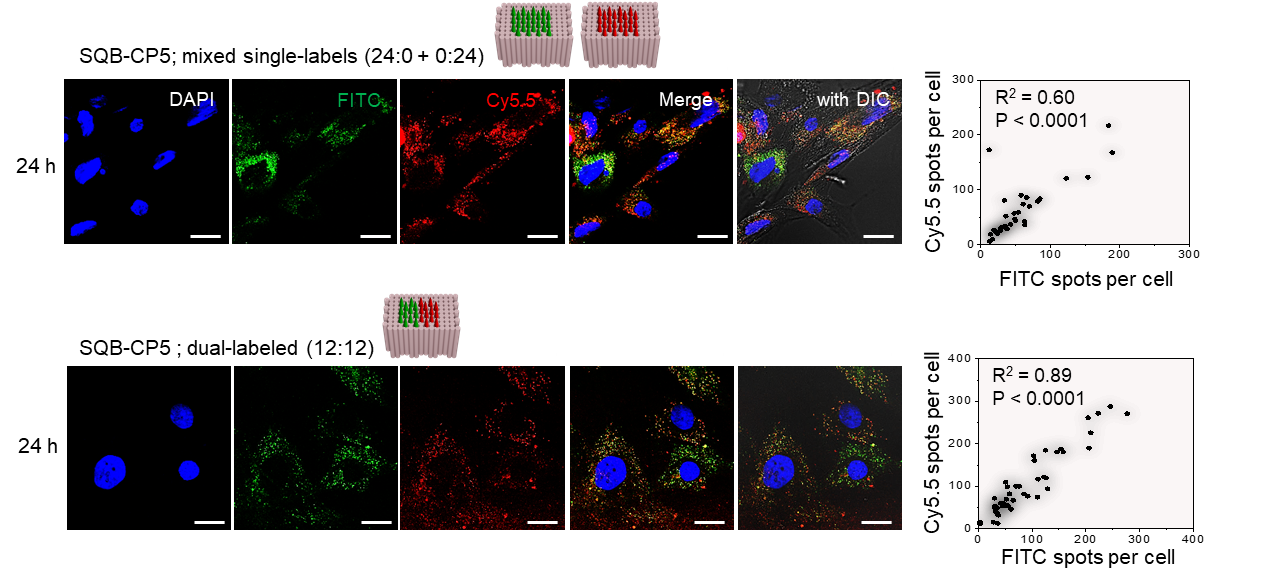


**Figure S12. Confocal imaging of within-cell co-delivery for singly mixed versus dually labeled SQB-CP5.** hMSCs were treated with a 1:1 mixture of singly labeled SQB-CP5s (24:0 + 0:24) (top) and a dually labeled SQB-CP5 (12:12) (bottom) and imaged at 24 h. Single-cell uptake quantified as spot counts per cell: FITC (x-axis) versus Cy5.5 (y-axis). R^2^ values are shown on the plots. Channels shown (left to right): DAPI, FITC, Cy5.5, Merge, and Merge with DIC. The scale bars indicate 20 μm.


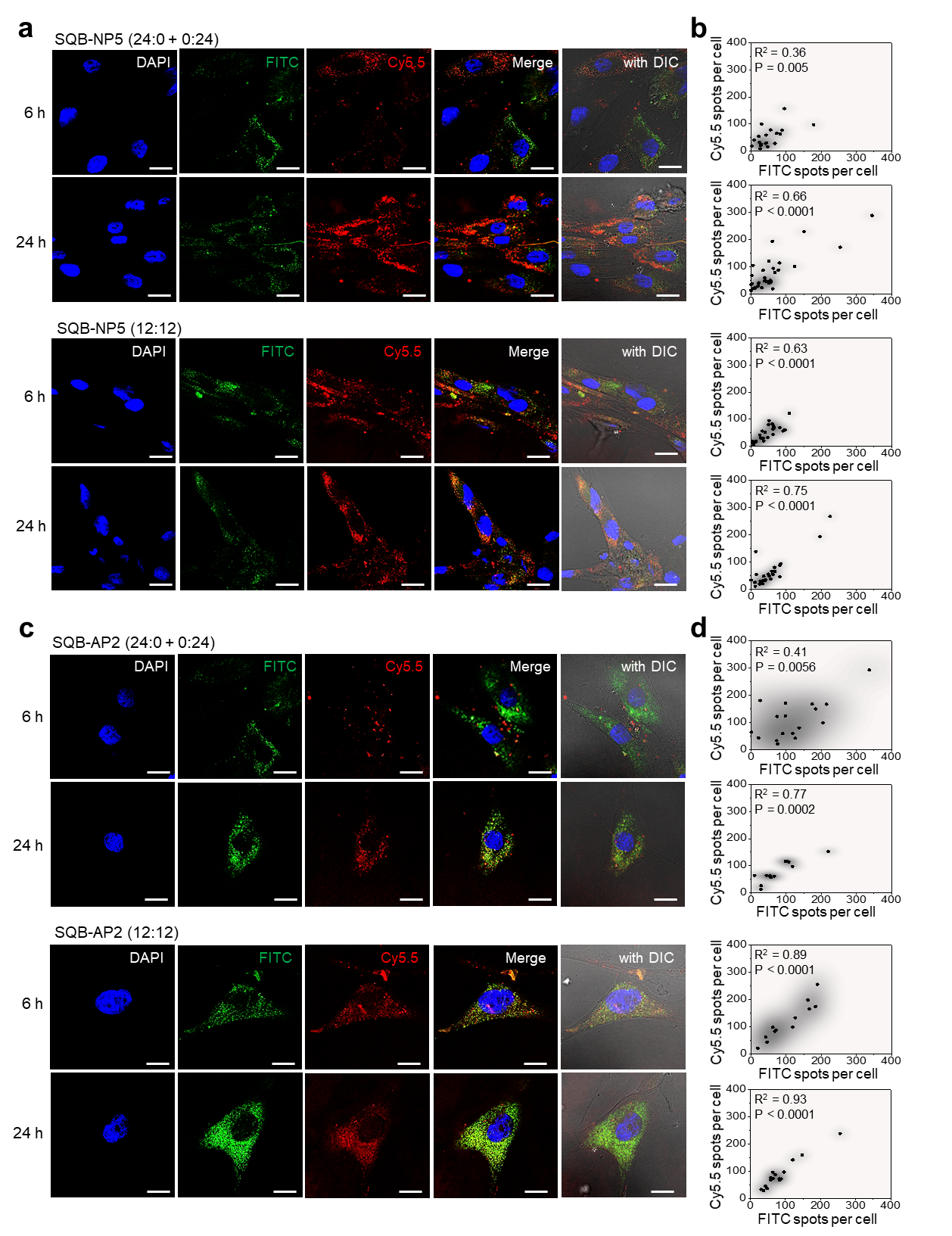


**Figure S13. Comprehensive co-delivery analysis for SQBs functionalized with neutral (NP5) and anionic (AP2) peptides.** This figure provides the comprehensive dataset for the co-delivery experiments using neutral and anionic peptides, supporting the findings presented in Figure 5. (a, b) Confocal microscopy images (a) and corresponding single-cell correlation analysis (b) for hMSCs treated with SQBs functionalized with the neutral peptide NP5 (SQB-NP5). The results demonstrate that the dual-labeled strategy leads to robust synchronized co-delivery, consistent with the findings for other peptide charge types. (c, d) The complete confocal imaging (c) and single-cell correlation analysis (d) for hMSCs treated with SQBs functionalized with the anionic peptide AP2 (SQB-AP2), including the 24-hour time point data which complements the representative data shown in the main Figure 5f, g. For all conditions, cells were treated with either a 1:1 mixture of singly labeled nanostructures (24:0 + 0:24) or with dual-labeled nanostructures (12:12). Channels shown: DAPI, FITC, Cy5.5, Merge, and Merge with DIC. Scale bars, 20 µm. For each hMSC, 15-20 cells were randomly selected for the correlation analysis. Correlation plots show FITC spots (x-axis) vs. Cy5.5 spots (y-axis); R² and P values are shown.

**
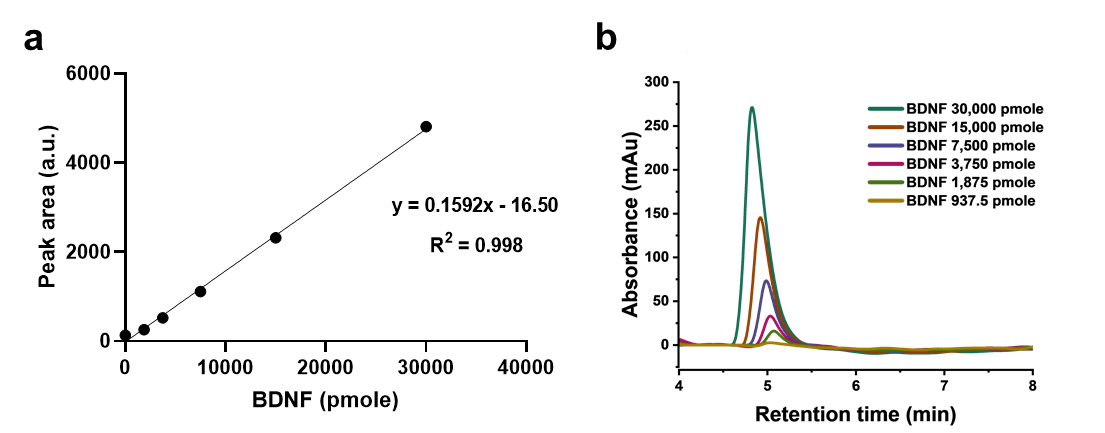
**

**Figure S14. Standard calibration curves, chromatographic identification and quantification of intact BDNF peptide by RP-HPLC.** (a) Standard calibration curve of the BDNF peptide obtained by RP-HPLC. Linear regression yielded y = 0.1592x – 16.50 (R^2^ = 0.998). (b) Overlaid chromatograms of the BDNF peptide standard. The eluent peak observed at 4.5-5.6 min. consistently exhibited the highest intensity and reproducibility across replicate injections, indicating that it represents the predominant intact peptide species under the applied chromatographic conditions. This peak was therefore considered to correspond to the intact peptide species and was selected as the reference peak for quantitative analysis. RP-HPLC quantification yielded 37.14 ± 2.26 peptides per SQB, indicating substantial non-specific adsorption. SQB was incubated with BDNF peptide lacking the complementary anti-handle at a 38× molar excess to SQB, which is onefold relative to the handle sites.

**Figure S15.** Zeta-potential of SQB and SQB^ads^-BDNF. Each dot represents one of the three different technical replicates. Data presented as mean ± SD (n=3).


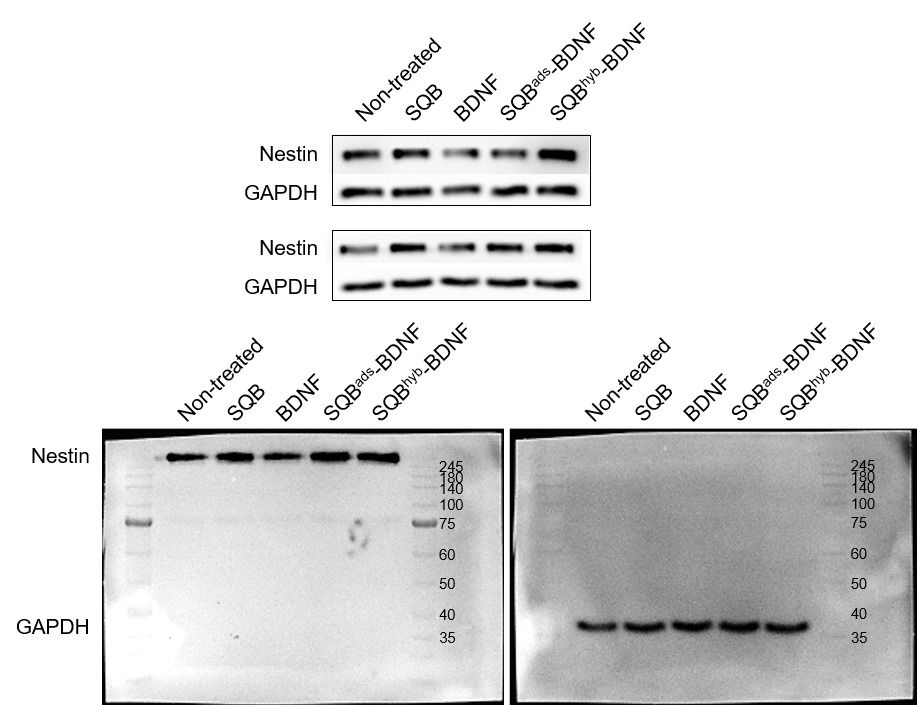


**Figure S16. Replicate western blot data for nestin expression analysis.** This figure displays the two additional, independent biological replicates for the Western blot analysis of nestin expression shown in the main Figure 6d. Human mesenchymal stem cells (hMSCs) were treated with the indicated formulations (Non-treated, SQB, BDNF, SQB^ads^-BDNF, and SQB^hyb^-BDNF), and cell lysates were probed for nestin (~280 kDa) and the loading control, GAPDH (~36 kDa). Together with the representative blot in the main figure, these data were used for the quantification presented in Figure 6e, confirming the reproducibility of the findings.

**Table S1. Anti-handle ssDNA employed for peptide and dye conjugation.** Sequences of the ssDNA anti-handles used for SPAAC-based conjugation with azide-functionalized peptides and for hybridization with complementary strands on DNA origami structures.

| **ssDNA sequence for peptide conjugates** | | **Complementary ssDNA sequence on SQB** |
| --- | --- | --- |
| ssDNA1 | GCTGTTAGAGAATGAGAGTCG | CGACTCTCATTCTCTAACAGC |
| ssDNA2 | AGTGATGTGAGACCATGTGAG | CTCACATGGTCTCACATCACT |
| ssDNA3 | TTCTAGGGTTAAAAGGGGACG | CGTCCCCTTTTAACCCTAGAA |

**Table S2. DNA scaffold for SQB (p8634 sequence).**

AATGCTACTACTATTAGTAGAATTGATGCCACCTTTTCAGCTCGCGCCCCAAATGAAAATATAGCTAAACAGGTTA TTGACCATTTGCGAAATGTATCTAATGGTCAAACTAAATCTACTCGTTCGCAGAATTGGGAATCAACTGTTATATG GAATGAAACTTCCAGACACCGTACTTTAGTTGCATATTTAAAACATGTTGAGCTACAGCATTATATTCAGCAATTA AGCTCTAAGCCATCCGCAAAAATGACCTCTTATCAAAAGGAGCAATTAAAGGTACTCTCTAATCCTGACCTGTTGG AGTTTGCTTCCGGTCTGGTTCGCTTTGAAGCTCGAATTAAAACGCGATATTTGAAGTCTTTCGGGCTTCCTCTTAAT CTTTTTGATGCAATCCGCTTTGCTTCTGACTATAATAGTCAGGGTAAAGACCTGATTTTTGATTTATGGTCATTCTCG TTTTCTGAACTGTTTAAAGCATTTGAGGGGGATTCAATGAATATTTATGACGATTCCGCAGTATTGGACGCTATCCA GTCTAAACATTTTACTATTACCCCCTCTGGCAAAACTTCTTTTGCAAAAGCCTCTCGCTATTTTGGTTTTTATCGTCG TCTGGTAAACGAGGGTTATGATAGTGTTGCTCTTACTATGCCTCGTAATTCCTTTTGGCGTTATGTATCTGCATTAG TTGAATGTGGTATTCCTAAATCTCAACTGATGAATCTTTCTACCTGTAATAATGTTGTTCCGTTAGTTCGTTTTATTA ACGTAGATTTTTCTTCCCAACGTCCTGACTGGTATAATGAGCCAGTTCTTAAAATCGCATAAGGTAATTCACAATG ATTAAAGTTGAAATTAAACCATCTCAAGCCCAATTTACTACTCGTTCTGGTGTTTCTCGTCAGGGCAAGCCTTATTC ACTGAATGAGCAGCTTTGTTACGTTGATTTGGGTAATGAATATCCGGTTCTTGTCAAGATTACTCTTGATGAAGGTC AGCCAGCCTATGCGCCTGGTCTGTACACCGTTCATCTGTCCTCTTTCAAAGTTGGTCAGTTCGGTTCCCTTATGATT GACCGTCTGCGCCTCGTTCCGGCTAAGTAACATGGAGCAGGTCGCGGATTTCGACACAATTTATCAGGCGATGATA CAAATCTCCGTTGTACTTTGTTTCGCGCTTGGTATAATCGCTGGGGGTCAAAGATGAGTGTTTTAGTGTATTCTTTT GCCTCTTTCGTTTTAGGTTGGTGCCTTCGTAGTGGCATTACGTATTTTACCCGTTTAATGGAAACTTCCTCATGAAA AAGTCTTTAGTCCTCAAAGCCTCTGTAGCCGTTGCTACCCTCGTTCCGATGCTGTCTTTCGCTGCTGAGGGTGACGA TCCCGCAAAAGCGGCCTTTAACTCCCTGCAAGCCTCAGCGACCGAATATATCGGTTATGCGTGGGCGATGGTTGTT GTCATTGTCGGCGCAACTATCGGTATCAAGCTGTTTAAGAAATTCACCTCGAAAGCAAGCTGATAAACCGATACAA TTAAAGGCTCCTTTTGGAGCCTTTTTTTTGGAGATTTTCAACGTGAAAAAATTATTATTCGCAATTCCTTTAGTTGTT CCTTTCTATTCTCACTCCGCTGAAACTGTTGAAAGTTGTTTAGCAAAATCCCATACAGAAAATTCATTTACTAACGT CTGGAAAGACGACAAAACTTTAGATCGTTACGCTAACTATGAGGGCTGTCTGTGGAATGCTACAGGCGTTGTAGTT TGTACTGGTGACGAAACTCAGTGTTACGGTACATGGGTTCCTATTGGGCTTGCTATCCCTGAAAATGAGGGTGGTG GCTCTGAGGGTGGCGGTTCTGAGGGTGGCGGTTCTGAGGGTGGCGGTACTAAACCTCCTGAGTACGGTGATACACC TATTCCGGGCTATACTTATATCAACCCTCTCGACGGCACTTATCCGCCTGGTACTGAGCAAAACCCCGCTAATCCTA ATCCTTCTCTTGAGGAGTCTCAGCCTCTTAATACTTTCATGTTTCAGAATAATAGGTTCCGAAATAGGCAGGGGGC ATTAACTGTTTATACGGGCACTGTTACTCAAGGCACTGACCCCGTTAAAACTTATTACCAGTACACTCCTGTATCAT CAAAAGCCATGTATGACGCTTACTGGAACGGTAAATTCAGAGACTGCGCTTTCCATTCTGGCTTTAATGAGGATTT ATTTGTTTGTGAATATCAAGGCCAATCGTCTGACCTGCCTCAACCTCCTGTCAATGCTGGCGGCGGCTCTGGTGGTG GTTCTGGTGGCGGCTCTGAGGGTGGTGGCTCTGAGGGTGGCGGTTCTGAGGGTGGCGGCTCTGAGGGAGGCGGTT CCGGTGGTGGCTCTGGTTCCGGTGATTTTGATTATGAAAAGATGGCAAACGCTAATAAGGGGGCTATGACCGAAA ATGCCGATGAAAACGCGCTACAGTCTGACGCTAAAGGCAAACTTGATTCTGTCGCTACTGATTACGGTGCTGCTAT CGATGGTTTCATTGGTGACGTTTCCGGCCTTGCTAATGGTAATGGTGCTACTGGTGATTTTGCTGGCTCTAATTCCC AAATGGCTCAAGTCGGTGACGGTGATAATTCACCTTTAATGAATAATTTCCGTCAATATTTACCTTCCCTCCCTCAA TCGGTTGAATGTCGCCCTTTTGTCTTTGGCGCTGGTAAACCATATGAATTTTCTATTGATTGTGACAAAATAAACTT ATTCCGTGGTGTCTTTGCGTTTCTTTTATATGTTGCCACCTTTATGTATGTATTTTCTACGTTTGCTAACATACTGCGT AATAAGGAGTCTTAATCATGCCAGTTCTTTTGGGTATTCCGTTATTATTGCGTTTCCTCGGTTTCCTTCTGGTAACTT TGTTCGGCTATCTGCTTACTTTTCTTAAAAAGGGCTTCGGTAAGATAGCTATTGCTATTTCATTGTTTCTTGCTCTTA TTATTGGGCTTAACTCAATTCTTGTGGGTTATCTCTCTGATATTAGCGCTCAATTACCCTCTGACTTTGTTCAGGGTG TTCAGTTAATTCTCCCGTCTAATGCGCTTCCCTGTTTTTATGTTATTCTCTCTGTAAAGGCTGCTATTTTCATTTTTGA CGTTAAACAAAAAATCGTTTCTTATTTGGATTGGGATAAATAATATGGCTGTTTATTTTGTAACTGGCAAATTAGGC TCTGGAAAGACGCTCGTTAGCGTTGGTAAGATTCAGGATAAAATTGTAGCTGGGTGCAAAATAGCAACTAATCTTG ATTTAAGGCTTCAAAACCTCCCGCAAGTCGGGAGGTTCGCTAAAACGCCTCGCGTTCTTAGAATACCGGATAAGCC TTCTATATCTGATTTGCTTGCTATTGGGCGCGGTAATGATTCCTACGATGAAAATAAAAACGGCTTGCTTGTTCTCG ATGAGTGCGGTACTTGGTTTAATACCCGTTCTTGGAATGATAAGGAAAGACAGCCGATTATTGATTGGTTTCTACA TGCTCGTAAATTAGGATGGGATATTATTTTTCTTGTTCAGGACTTATCTATTGTTGATAAACAGGCGCGTTCTGCAT TAGCTGAACATGTTGTTTATTGTCGTCGTCTGGACAGAATTACTTTACCTTTTGTCGGTACTTTATATTCTCTTATTA CTGGCTCGAAAATGCCTCTGCCTAAATTACATGTTGGCGTTGTTAAATATGGCGATTCTCAATTAAGCCCTACTGTT GAGCGTTGGCTTTATACTGGTAAGAATTTGTATAACGCATATGATACTAAACAGGCTTTTTCTAGTAATTATGATTC CGGTGTTTATTCTTATTTAACGCCTTATTTATCACACGGTCGGTATTTCAAACCATTAAATTTAGGTCAGAAGATGA AATTAACTAAAATATATTTGAAAAAGTTTTCTCGCGTTCTTTGTCTTGCGATTGGATTTGCATCAGCATTTACATAT

AGTTATATAACCCAACCTAAGCCGGAGGTTAAAAAGGTAGTCTCTCAGACCTATGATTTTGATAAATTCACTATTG ACTCTTCTCAGCGTCTTAATCTAAGCTATCGCTATGTTTTCAAGGATTCTAAGGGAAAATTAATTAATAGCGACGAT TTACAGAAGCAAGGTTATTCACTCACATATATTGATTTATGTACTGTTTCCATTAAAAAAGGTAATTCAAATGAAA TTGTTAAATGTAATTAATTTTGTTTTCTTGATGTTTGTTTCATCATCTTCTTTTGCTCAGGTAATTGAAATGAATAAT TCGCCTCTGCGCGATTTTGTAACTTGGTATTCAAAGCAATCAGGCGAATCCGTTATTGTTTCTCCCGATGTAAAAGG

TACTGTTACTGTATATTCATCTGACGTTAAACCTGAAAATCTACGCAATTTCTTTATTTCTGTTTTACGTGCAAATAA TTTTGATATGGTAGGTTCTAACCCTTCCATTATTCAGAAGTATAATCCAAACAATCAGGATTATATTGATGAATTGC CATCATCTGATAATCAGGAATATGATGATAATTCCGCTCCTTCTGGTGGTTTCTTTGTTCCGCAAAATGATAATGTT ACTCAAACTTTTAAAATTAATAACGTTCGGGCAAAGGATTTAATACGAGTTGTCGAATTGTTTGTAAAGTCTAATA

CTTCTAAATCCTCAAATGTATTATCTATTGACGGCTCTAATCTATTAGTTGTTAGTGCTCCTAAAGATATTTTAGAT AACCTTCCTCAATTCCTTTCAACTGTTGATTTGCCAACTGACCAGATATTGATTGAGGGTTTGATATTTGAGGTTCA GCAAGGTGATGCTTTAGATTTTTCATTTGCTGCTGGCTCTCAGCGTGGCACTGTTGCAGGCGGTGTTAATACTGACC GCCTCACCTCTGTTTTATCTTCTGCTGGTGGTTCGTTCGGTATTTTTAATGGCGATGTTTTAGGGCTATCAGTTCGCG CATTAAAGACTAATAGCCATTCAAAAATATTGTCTGTGCCACGTATTCTTACGCTTTCAGGTCAGAAGGGTTCTATC TCTGTTGGCCAGAATGTCCCTTTTATTACTGGTCGTGTGACTGGTGAATCTGCCAATGTAAATAATCCATTTCAGAC GATTGAGCGTCAAAATGTAGGTATTTCCATGAGCGTTTTTCCTGTTGCAATGGCTGGCGGTAATATTGTTCTGGATA TTACCAGCAAGGCCGATAGTTTGAGTTCTTCTACTCAGGCAAGTGATGTTATTACTAATCAAAGAAGTATTGCTAC AACGGTTAATTTGCGTGATGGACAGACTCTTTTACTCGGTGGCCTCACTGATTATAAAAACACTTCTCAGGATTCTG GCGTACCGTTCCTGTCTAAAATCCCTTTAATCGGCCTCCTGTTTAGCTCCCGCTCTGATTCTAACGAGGAAAGCACG TTATACGTGCTCGTCAAAGCAACCATAGTACGCGCCCTGTAGCGGCGCATTAAGCGCGGCGGGTGTGGTGGTTACG CGCAGCGTGACCGCTACACTTGCCAGCGCCCTAGCGCCCGCTCCTTTCGCTTTCTTCCCTTCCTTTCTCGCCACGTTC GCCGGCTTTCCCCGTCAAGCTCTAAATCGGGGGCTCCCTTTAGGGTTCCGATTTAGTGCTTTACGGCACCTCGACCC CAAAAAACTTGATTTGGGTGATGGTTCACGTAGTGGGCCATCGCCCTGATAGACGGTTTTTCGCCCTTTGACGTTG GAGTCCACGTTCTTTAATAGTGGACTCTTGTTCCAAACTGGAACAACACTCAACCCTATCTCGGGCTATTCTTTTGA TTTATAAGGGATTTTGCCGATTTCGGAACCACCATCAAACAGGATTTTCGCCTGCTGGGGCAAACCAGCGTGGACC GCTTGCTGCAACTCTCTCAGGGCCAGGCGGTGAAGGGCAATCAGCTGTTGCCCGTCTCACTGGTGAAAAGAAAAA CCACCCTGGCGCCCAATACGCAAACCGCCTCTCCCCGCGCGTTGGCCGATTCATTAATGCAGCTGGCACGACAGGT TTCCCGACTGGAAAGCGGGCAGTGAGCGCAACGCAATTAATGTGAGTTAGCTCACTCATTAGGCACCCCAGGCTTT ACACTTTATGCTTCCGGCTCGTATGTTGTGTGGAATTGTGAGCGGATAACAATTTCACACAGGAAACAGCTATGAC CATGATTACGAATTCGAGCTCGGTACCCGGGGATCCATTCTCCTGTGACTCGGAAGTGCATTTATCATCTCCATAA AACAAAACCCGCCGTAGCGAGTTCAGATAAAATAAATCCCCGCGAGTGCGAGGATTGTTATGTAATATTGGGTTTA ATCATCTATATGTTTTGTACAGAGAGGGCAAGTATCGTTTCCACCGTACTCGTGATAATAATTTTGCACGGTATCAG TCATTTCTCGCACATTGCAGAATGGGGATTTGTCTTCATTAGACTTATAAACCTTCATGGAATATTTGTATGCCGAC TCTATATCTATACCTTCATCTACATAAACACCTTCGTGATGTCTGCATGGAGACAAGACACCGGATCTGCACAACA TTGATAACGCCCAATCTTTTTGCTCAGACTCTAACTCATTGATACTCATTTATAAACTCCTTGCAATGTATGTCGTTT CAGCTAAACGGTATCAGCAATGTTTATGTAAAGAAACAGTAAGATAATACTCAACCCGATGTTTGAGTACGGTCAT CATCTGACACTACAGACTCTGGCATCGCTGTGAAGACGACGCGAAATTCAGCATTTTCACAAGCGTTATCTTTTAC AAAACCGATCTCACTCTCCTTTGATGCGAATGCCAGCGTCAGACATCATATGCAGATACTCACCTGCATCCTGAAC CCATTGACCTCCAACCCCGTAATAGCGATGCGTAATGATGTCGATAGTTACTAACGGGTCTTGTTCGATTAACTGC CGCAGAAACTCTTCCAGGTCACCAGTGCAGTGCTTGATAACAGGAGTCTTCCCAGGATGGCGAACAACAAGAAAC TGGTTTCCGTCTTCACGGACTTCGTTGCTTTCCAGTTTAGCAATACGCTTACTCCCATCCGAGATAACACCTTCGTA ATACTCACGCTGCTCGTTGAGTTTTGATTTTGCTGTTTCAAGCTCAACACGCAGTTTCCCTACTGTTAGCGCAATAT CCTCGTTCTCCTGGTCGCGGCGTTTGATGTATTGCTGGTTTCTTTCCCGTTCATCCAGCAGTTCCAGCACAATCGAT GGTGTTACCAATTCATGGAAAAGGTCTGCGTCAAATCCCCAGTCGTCATGCATTGCCTGCTCTGCCGCTTCACGCA GTGCCTGAGAGTTAATTTCGCTCACTTCGAACCTCTCTGTTTACTGATAAGTTCCAGATCCTCCTGGCAACTTGCAC AAGTCCGACAACCCTGAACGACCAGGCGTCTTCGTTCATCTATCGGATCGCCACACTCACAACAATGAGTGGCAG ATATAGCCTGGTGGTTCAGGCGGCGCATTTTTATTGCTGTGTTGCGCTGTAATTCTTCTATTTCTGATGCTGAATCA ATGATGTCTGCCATCTTTCATTAATCCCTGAACTGTTGGTTAATACGCATGAGGGTGAATGCGAATAATAAAGCTT GGCACTGGCCGTCGTTTTACAACGTCGTGACTGGGAAAACCCTGGCGTTACCCAACTTAATCGCCTTGCAGCACAT CCCCCTTTCGCCAGCTGGCGTAATAGCGAAGAGGCCCGCACCGATCGCCCTTCCCAACAGTTGCGCAGCCTGAATG GCGAATGGCGCTTTGCCTGGTTTCCGGCACCAGAAGCGGTGCCGGAAAGCTGGCTGGAGTGCGATCTTCCTGAGGC CGATACTGTCGTCGTCCCCTCAAACTGGCAGATGCACGGTTACGATGCGCCCATCTACACCAACGTGACCTATCCC ATTACGGTCAATCCGCCGTTTGTTCCCACGGAGAATCCGACGGGTTGTTACTCGCTCACATTTAATGTTGATGAAA GCTGGCTACAGGAAGGCCAGACGCGAATTATTTTTGATGGCGTTCCTATTGGTTAAAAAATGAGCTGATTTAACAA AAATTTAATGCGAATTTTAACAAAATATTAACGTTTACAATTTAAATATTTGCTTATACAATCTTCCTGTTTTTGGG GCTTTTCTGATTATCAACCGGGGTACATATGATTGACATGCTAGTTTTACGATTACCGTTCATCGATTCTCTTGTTTG CTCCAGACTCTCAGGCAATGACCTGATAGCCTTTGTAGATCTCTCAAAAATAGCTACCCTCTCCGGCATTAATTTAT CAGCTAGAACGGTTGAATATCATATTGATGGTGATTTGACTGTCTCCGGCCTTTCTCACCCTTTTGAATCTTTACCT ACACATTACTCAGGCATTGCATTTAAAATATATGAGGGTTCTAAAAATTTTTATCCTTGCGTTGAAATAAAGGCTTC TCCCGCAAAAGTATTACAGGGTCATAATGTTTTTGGTACAACCGATTTAGCTTTATGCTCTGAGGCTTTATTGCTTA

ATTTTGCTAATTCTTTGCCTTGCCTGTATGATTTATTGGATGTT

**Table S3. Staple sequence for bare SQB.**

| Name | Sequence | Length and positions |
| --- | --- | --- |
| Oligo0 | TAAATCGGTATCAATGAGAACCCTAATTGAGGAAGACTCCAATTTCAT | 48mer, [3, 64] start, [48, 64] end |
| Oligo1 | AAAGGGAGTTTTTCTTAATAAAAGGATATAGAAACCAGTT | 40mer, [4, 71] start, [34, 64] end |
| Oligo2 | ACCACACCCCTGGGGTAACTATCGGATACTTGTATTGCGC | 40mer, [8, 71] start, [38, 64] end |
| Oligo3 | CCGATTAACTGTACAAGTGTTTTTTCAATATAGGGGATTT | 40mer, [11, 64] start, [41, 71] end |
| Oligo4 | TGAGGCCACTCGAATTCGTAAGATTTTAGACAATGATAAA | 40mer, [15, 80] start, [13, 71] end |
| Oligo5 | AGAACTCAGCCTAATGTCCACACAACATACGA | 32mer, [16, 79] start, [10, 64] end |
| Oligo6 | CAATATTAAAATCCTTTGAGCTTGTACATCGGGTCGGACTTCGTAAAA | 48mer, [19, 64] start, [66, 64] end |
| Oligo7 | CGACCAGTTTCACCAGGCGGGGAGAGGCGGTTCGCTGCGCATACAAAT | 48mer, [20, 79] start, [18, 64] end |
| Oligo8 | AGTTTATAGTCAATGGCAACAGTTTAAAACGAAACAGTACAAATTTAA | 48mer, [25, 64] start, [60, 64] end |
| Oligo9 | AGGCGGTCAGTATATATACCGAACAGAATAGCTGTTTGATGGTGGTTC | 48mer, [27, 64] start, [2, 64] end |
| Oligo10 | ACGCTTGTCAAGAGTCCACTATTAAAGAACGTAAATCAAAGAACCACC | 48mer, [27, 80] start, [24, 64] end |
| Oligo11 | AAGTAACACCGCCTGCTGCATATGGAAGAGTC | 32mer, [28, 79] start, [52, 64] end |
| Oligo12 | AGCACTGCCAGTCACAGAAAGCGTTGGCCCTGAGAGACCT | 40mer, [33, 80] start, [4, 72] end |
| Oligo13 | CGACAACTCGCAACACTGATTGCTTAAAGCCA | 32mer, [35, 64] start, [64, 64] end |
| Oligo14 | AAATCAAATGAGTAGACATTGCAACTGCCCGCTTTCCACC | 40mer, [37, 80] start, [8, 72] end |
| Oligo15 | ATGATGGCAGTTGCCAGAATAATGGTAATTCTCAACCGTT | 40mer, [39, 64] start, [69, 71] end |
| Oligo16 | AGAACCTACTGGAACTGCTGGTCAATAATCAG | 32mer, [43, 80] start, [15, 79] end |
| Oligo17 | AAAGAGCAAATATGAACGGGAAAGCAAATTAA | 32mer, [42, 79] start, [14, 80] end |
| Oligo18 | ACCTGCAGCGTGGATGGGAGTAAGACATTTTG | 32mer, [46, 79] start, [18, 80] end |
| Oligo19 | TAGAAGAATTTAACCAAATTCTTAGCAAAGAAAGTACCGCAGGAAACC | 48mer, [49, 64] start, [92, 64] end |
| Oligo20 | TTAATTTGGAACAAGACCCGTTAGTTGAATGG | 32mer, [50, 79] start, [22, 80] end |
| Oligo21 | CAGTCACGGGTGTAGATCATCTTCTAGATACAAGGTTTTGGAGAGATA | 48mer, [53, 64] start, [88, 64] end |
| Oligo22 | ACGTAATTTTCCCTTATATTACGGCGCTGGCAATTAAAAA | 40mer, [53, 72] start, [24, 80] end |
| Oligo23 | TAGGTCTGAGAGATCCGACGCTGAATGTCTGAGGTTGGAGAATAGTCT | 48mer, [55, 64] start, [25, 79] end |
| Oligo24 | CTTCTACCTTTTTAACCTGCCAGTAAACAGCC | 32mer, [56, 79] start, [80, 64] end |
| Oligo25 | ACGACGACTAGATTAAGGCACCGCTTTTGTAA | 32mer, [57, 80] start, [28, 80] end |
| Oligo26 | ACCCGCCTATAAATCACATTTAACTGTTATCAAAGACGGAGTCCAATC | 48mer, [60, 79] start, [21, 79] end |
| Oligo27 | TTCGCGTCGGCGTTTTATCATACAGCGGAATCACAAAGTTCCAGGCGC | 48mer, [61, 64] start, [102, 64] end |
| Oligo28 | CTGTAGCCATTAATTAATATATGTGCAGTTAATCGAAAGGTCTGACCT | 48mer, [61, 80] start, [23, 79] end |
| Oligo29 | ATTGTGTATTGAATACGTACCTTTAAACAGCAAACGAGGACCCGTAGC | 48mer, [64, 79] start, [17, 79] end |
| Oligo30 | CCCGGTTGCTTATCATGCAAGGATGGAATTAC | 32mer, [65, 64] start, [94, 64] end |
| Oligo31 | AAAAGCCCCAGTAACACAAGTTACAAGGTGTTATCTCATTCCGCCAGC | 48mer, [65, 80] start, [19, 79] end |
| Oligo32 | CGACAAAATTTTAGAAGTTCAGCTACGCAAAGAAGAAAAA | 40mer, [67, 64] start, [97, 71] end |
| Oligo33 | ACGCGCCTAGATCTACAAAGGAAAGAAGGGTT | 32mer, [71, 80] start, [43, 79] end |
| Oligo34 | AATAAAATGGTCTATCAGGTCATTACAGAAAT | 32mer, [70, 79] start, [42, 80] end |
| Oligo35 | TGTCTTTCATAATCAGAACGGTAATGTAATTGCGTAGATTACCAGGAG | 48mer, [72, 79] start, [38, 80] end |
| Oligo36 | GAACGCGATGGCCTTCGCTCATTTTTAAGCAAAAGAAGATAGTCCGTG | 48mer, [76, 79] start, [34, 80] end |
| Oligo37 | CCTGACATTGATCGGATTCTCCGTCGTCGCTA | 32mer, [78, 79] start, [50, 80] end |
| Oligo38 | TTTAGTTTGCCCGAAAGAGCGCTACCTGATAACGGTCATAATGGCTTT | 48mer, [81, 64] start, [116, 64] end |
| Oligo39 | ATTGCTGATAAGAAACGATTTCTTTACAAAATTTGAGGGGGTCACGTT | 48mer, [82, 71] start, [58, 72] end |
| Oligo40 | TTAACGTCTTCGAGCTGCCACCACGATTTGTAGCAGGGAG | 40mer, [84, 71] start, [114, 64] end |
| Oligo41 | AAGAGGAAGACATCTTACCAACGCTGGGATAG | 32mer, [86, 79] start, [58, 80] end |
| Oligo42 | CCCTCAAACAGACTGTGAACGGTGAAAAGGCTTATTCTGAAACGGAAC | 48mer, [89, 64] start, [119, 79] end |
| Oligo43 | TGCCAGAGAATCATTACCGCGAAGCCAAAACGTTAATATTTTTCAATT | 48mer, [91, 80] start, [46, 80] end |
| Oligo44 | ATCATAACCCGTCACCGACGAGAAGTTTTGTCTAGGTGTATCATACAA | 48mer, [93, 64] start, [123, 79] end |
| Oligo45 | AACATATAGAACGAGTACAATCAATACTCAGGAGGGATAG | 40mer, [95, 64] start, [125, 71] end |
| Oligo46 | TCATATGGGTAGAAAGATTCAGAAAATGCAGA | 32mer, [99, 80] start, [71, 79] end |
| Oligo47 | GGAGGGAAATACATAAACCAGACGTAATCGGCAGAACAAGATATTTAA | 48mer, [101, 80] start, [64, 80] end |
| Oligo48 | AATTATCACCTCGTTTCGCCAAAAAAATATCCCATCCTAAAATCGATG | 48mer, [100, 79] start, [66, 80] end |
| Oligo49 | TAAAGGTGCAGTAGCACTTTTGCAAAAGAAGAACTCATCG | 40mer, [100, 87] start, [75, 79] end |
| Oligo50 | GAGCCAGCAAAGTATTGAATTTTCTGTATGGGGATCTAAAACATTGAG | 48mer, [103, 64] start, [101, 79] end |
| Oligo51 | GATAGCAGGATAGCGTACAGTTCATATTCTAAAATCAAGAGAGTAACA | 48mer, [105, 80] start, [60, 80] end |
| Oligo52 | TTTAGCGTTGCTTTAACCAATACTGGCCCCAATAGCAAGCTTAAATCA | 48mer, [104, 79] start, [62, 80] end |
| Oligo53 | AGTTTGCCATTAGCGTAATCAGGTCTTTATCAAAGCCTTA | 40mer, [104, 87] start, [79, 79] end |
| Oligo54 | AGAGCTCGATACCCTGACTATTATAATTTTAT | 32mer, [106, 79] start, [78, 80] end |
| Oligo55 | CCGGAACCAAAAGATTAACCAGACTTGCCAGTTAATTGCTCCAGCCAG | 48mer, [109, 80] start, [56, 80] end |
| Oligo56 | AACCGCCAGACTAAAGTTAGAGAGTACTTTGT | 32mer, [108, 87] start, [84, 72] end |
| Oligo57 | AATGCCACACCACCAGAGCCGGAGCCCTCAGATCAAAGCG | 40mer, [110, 71] start, [85, 79] end |
| Oligo58 | AGCATTGACGTCACCCTCAGCAGCTGAGGCTTTCACACCA | 40mer, [112, 71] start, [109, 79] end |
| Oligo59 | AGTAAGCGTCAACAATGACAACAACCGGAACC | 32mer, [115, 64] start, [106, 80] end |
| Oligo60 | TCGGTTTATCAGCTTGCTCCAAAATACCCATC | 32mer, [117, 64] start, [105, 79] end |
| Oligo61 | AACTAAAGGTCACCAATGAAAAGAACCAGTTT | 32mer, [119, 80] start, [91, 79] end |
| Oligo62 | CGCCTGTAGACATTCAACCGACCAAAATCAGTTGAGATTTACAAGAAA | 48mer, [123, 80] start, [70, 80] end |
| Oligo63 | GGACTCCACAGAGGTGGTCAGATGATGACCGTACTCAAAC | 40mer, [0, 63] start, [26, 40] end |
| Oligo64 | TTTTTTTTTTGGGCGATGGCCCCATAAACATTGCTTTTTTTTTT | 44mer, [1, 17] start, [24, 17] end |
| Oligo65 | AGGTGCCGTAAAGCACCGAAATCGTTGCAAGGAGCAGAAG | 40mer, [3, 48] start, [24, 56] end |
| Oligo66 | TTTTTTTTTTCGTGGCGAGAAATTGTCTCCATGCTTTTTTTTTT | 44mer, [5, 17] start, [20, 17] end |
| Oligo67 | AGAAACGCCAGGGTGGCCCCCGATACAGAGATAGTTAGAGTGGCAAAT | 48mer, [5, 48] start, [31, 63] end |
| Oligo68 | GCAAGTGTAGCGGTCATGCGTATTAAGGTATAGGACATTCATCCTGGG | 48mer, [7, 48] start, [33, 63] end |
| Oligo69 | TTTTTTTTTTTGGTTGCTTTGAGAAATGACTGATTTTTTTTTTT | 44mer, [9, 17] start, [16, 17] end |
| Oligo70 | TATAAAAAGTGTAAAGCGCCGCGCATCCAGAAATTCCATG | 40mer, [9, 48] start, [18, 56] end |
| Oligo71 | GGAACGGTACGCCGCAGGAGCTAAACAGGAGGGCCGGAAGGTGGAAAC | 48mer, [12, 63] start, [17, 63] end |
| Oligo72 | TTTTTTTTTTTCTGAACTCGCTACGGCGGGTTTTGTTTTATGGAG | 45mer, [13, 17] start, [13, 63] end |
| Oligo73 | CTCAGAATCCTGAGAAAACATATAATTATCAGTAACAGTATCAGGGTT | 48mer, [15, 48] start, [45, 63] end |
| Oligo74 | AACCCAATTCAGAGCGCTCGCGGGGATTTATTTTATTTTTTTTTT | 45mer, [14, 47] start, [12, 17] end |
| Oligo75 | TTTTTTTTTTACCGTGCAAAATAGAAACCACCAGTTTTTTTTTT | 44mer, [17, 17] start, [38, 17] end |
| Oligo76 | GCCTTGCTCTGCGTGTTGCCCGAAGATTCGCC | 32mer, [16, 63] start, [47, 63] end |
| Oligo77 | TAAGTCTAGGGCGCTGCCGCTACAGGGCGCGTACTATTTTTTTTTT | 46mer, [18, 47] start, [8, 17] end |
| Oligo78 | TTTTTTTTTTAGACATCACGAATAGATAATACATTTTTTTTTTT | 44mer, [21, 17] start, [34, 17] end |
| Oligo79 | AAAAGATTTGGGGTCGTGACGGGGAAAGCCGGCGAATTTTTTTTTT | 46mer, [22, 47] start, [4, 17] end |
| Oligo80 | TTTTTTTTTTTGATACCGTTTAGAACCTCAAATATTTTTTTTTT | 44mer, [25, 17] start, [30, 17] end |
| Oligo81 | TTTCTTTAACTACGTGAACCAAAATCCCTTAT | 32mer, [24, 47] start, [1, 63] end |
| Oligo82 | TATCTAAAACGTCAAAGGGCGAAAAACCGTCTATCATTTTTTTTTT | 46mer, [27, 48] start, [0, 17] end |
| Oligo83 | TGAGTATCAACAGTGCCAAAATCAACTGTTGG | 32mer, [29, 56] start, [54, 56] end |
| Oligo84 | GTTCAGGAGGTTTTCCAATAGTGACCGTGCAT | 32mer, [30, 63] start, [57, 63] end |
| Oligo85 | TTTTTTTTTTTCAAACCCTCAACGCATTCACCCTTTTTTTTTTT | 44mer, [31, 17] start, [50, 17] end |
| Oligo86 | TGGTCAGTTCTGAGCAGACATACAGCATCACCCAAATCAA | 40mer, [31, 48] start, [2, 40] end |
| Oligo87 | AAGGTTATTTAATGGACGGCCAGTAGTTAATTTGGGCGCA | 40mer, [32, 63] start, [58, 56] end |
| Oligo88 | ATAGAGCCTGGCAGATCCGGTGTCGGAAGGGA | 32mer, [33, 48] start, [5, 47] end |
| Oligo89 | TTTTTTTTTTTTGAGGATTTAGCCACCAGGCTATTTTTTTTTTT | 44mer, [35, 17] start, [46, 17] end |
| Oligo90 | GACTTTACAAGGTTTATGTAGATGGGGGCGAAAGGAGCGG | 40mer, [35, 48] start, [6, 40] end |
| Oligo91 | AAACAATTTCTTGTTGATCAGAAATTGAATTAAAAAATAATGGTTTGA | 48mer, [35, 56] start, [60, 56] end |
| Oligo92 | TATCAAAAGGTCTGCAATGTGCGACGAGCACG | 32mer, [37, 48] start, [9, 47] end |
| Oligo93 | TTTTTTTTTTAAGGAGCGGAATACAGAGAGGTTCTTTTTTTTTT | 44mer, [39, 17] start, [42, 17] end |
| Oligo94 | TATTCCTGGATGATTACGAGTACGCATCGTGCTTTCCTCG | 40mer, [39, 48] start, [10, 40] end |
| Oligo95 | CAGAGCAGGCAATGCATGACGACTATCCTGATTGTTTGGC | 40mer, [41, 40] start, [40, 48] end |
| Oligo96 | TTTTTTTTTTGAAGTGAGCGAAAAGGGTGAGAAATTTTTTTTTT | 44mer, [43, 17] start, [68, 17] end |
| Oligo97 | TCAGGATTATACTTCTGGAGGATCTAAAGTACCTAGCATGATTTCAAC | 48mer, [43, 48] start, [73, 63] end |
| Oligo98 | CTGGTCGTGGGTTTTGCGGAACAATATTATCA | 32mer, [45, 48] start, [17, 47] end |
| Oligo99 | GAGAAACAATATGTACACGCTCAATTAAACCA | 32mer, [44, 63] start, [75, 63] end |
| Oligo100 | TTTTTTTTTTATCTGCCACTCAAGAATCGCCATATTTTTTTTTT | 44mer, [47, 17] start, [64, 17] end |
| Oligo101 | CGCCTGAAAAGTATTAATTTTAAAACAAATCCCCATTAATTTAATGCG | 48mer, [46, 47] start, [8, 48] end |
| Oligo102 | AGCAATAAGTTATACAATAGGAACTCCAATAA | 32mer, [46, 63] start, [77, 63] end |
| Oligo103 | TTTTTTTTTTCATGCGTATTAATAAATAAGGCGTTTTTTTTTTT | 44mer, [51, 17] start, [60, 17] end |
| Oligo104 | TTATTATTTCAATATCTCTTTAGGTATCAATGTTGTGCCATTAGAGCT | 48mer, [50, 47] start, [4, 48] end |
| Oligo105 | CTGCAAGGTGAAAAATCTAAAAGGATATACTG | 32mer, [52, 47] start, [24, 48] end |
| Oligo106 | TTTTTTTTTTTATTACGCCAGCACTATATGTAAATTTTTTTTTT | 44mer, [55, 17] start, [56, 17] end |
| Oligo107 | GGGGGTATCACGCTGAGAGCCAGCTTGAGTAT | 32mer, [55, 48] start, [27, 47] end |
| Oligo108 | GAAGGGCGATCGGTGCGGGCCTCTTCGCTTTTTTTTTT | 38mer, [54, 55] start, [54, 17] end |
| Oligo109 | CTCCGGCTAATCCAAAATATAATGCTGTAGCTCAACATGT | 40mer, [56, 63] start, [82, 40] end |
| Oligo110 | TTTTTTTTTTTGCTGATGCAAAAGTTTCATTCCATTTTTTTTTT | 44mer, [57, 17] start, [80, 17] end |
| Oligo111 | TATATTTTGCCAAGCTAACGCCAGTGCGCATCACCTTGCTGCTGAAAC | 48mer, [59, 48] start, [25, 47] end |
| Oligo112 | TTTTTTTTTTTAAATAAGAATAAAAGGTGGCATCTTTTTTTTTT | 44mer, [61, 17] start, [76, 17] end |
| Oligo113 | AATCAATCCCTGATTAATGAAAGAAACAACTA | 32mer, [61, 48] start, [33, 47] end |
| Oligo114 | TCATATGCAAATGCGCGATTCAGCTTCTTAGAGCCGTCAAGGTGTTTA | 48mer, [63, 48] start, [21, 47] end |
| Oligo115 | AGGCATTTGAAGACGCATCAGTAATATCATCAACTGCGTGTAACAATC | 48mer, [66, 47] start, [15, 47] end |
| Oligo116 | TTTTTTTTTTTTTAACAACGCCAACATTATGACCTTTTTTTTTT | 44mer, [65, 17] start, [72, 17] end |
| Oligo117 | ATTTAATCATAGGCGATCCGATAGAGTAACAT | 32mer, [65, 48] start, [37, 47] end |
| Oligo118 | TTTTTTTTTTGGCCGGAGACAGTCAAATCACCATCAAT | 38mer, [69, 17] start, [69, 55] end |
| Oligo119 | ATGATATTGTCCAGACGACGATAAAGAGAATATGGAACTT | 40mer, [69, 56] start, [42, 48] end |
| Oligo120 | AGGCAATAAACAACATCCCTCATAAAGGTGGCGAGGCATACTTGCCCT | 48mer, [71, 48] start, [101, 63] end |
| Oligo121 | ATGCAATGCAGTAATAAGATTCAAATTAACTC | 32mer, [70, 47] start, [43, 47] end |
| Oligo122 | TTTTTTTTTTCTGTAATACTTTATTACGCAGTATTTTTTTTTTT | 44mer, [73, 17] start, [94, 17] end |
| Oligo123 | TCCAAGAAGCAACACTGAGGAAACGGGAATTAATAGGCTGGAACCTAT | 48mer, [72, 63] start, [119, 63] end |
| Oligo124 | ATAAAGCCGTTTAGTACTTAATTGTTGTTGTGAGTGTACGCGTTATTA | 48mer, [74, 47] start, [36, 48] end |
| Oligo125 | ATTAAGCACATTAACAGCCTAATTACTAGAAAACATCATT | 40mer, [74, 55] start, [49, 47] end |
| Oligo126 | TTAGCAAAATAGCCGAGTCATAAAGGACAGAT | 32mer, [74, 63] start, [105, 63] end |
| Oligo127 | TTTTTTTTTTAATTCTACTAATTAGCTATCTTACTTTTTTTTTT | 44mer, [77, 17] start, [90, 17] end |
| Oligo128 | AGCGAACCTTGAATCCACCCACAAGGCATTTT | 32mer, [76, 63] start, [107, 63] end |
| Oligo129 | CTTGCGGGTTTCGCAAGGGTAATTGACTTCAAACAACGGA | 40mer, [79, 56] start, [109, 63] end |
| Oligo130 | TAACCTGTTTTTCAAACCGTGTGACCAACAGTTCAGGTTTCTAAAATA | 48mer, [78, 47] start, [32, 48] end |
| Oligo131 | ATGGTCAATCTGCGAATCGCAAAGAACGCGAGAGTTGGGT | 40mer, [78, 55] start, [53, 47] end |
| Oligo132 | TTTTTTTTTTTATAACAGTTGATAGACGGGAGAATTTTTTTTTT | 44mer, [81, 17] start, [86, 17] end |
| Oligo133 | CGAGTAGAATATTATTCGTTTTAAAAAAATGACCAGAACCTACGAAGG | 48mer, [81, 56] start, [110, 56] end |
| Oligo134 | TGTCTGGATCCAATCGCAAGATAAATTATGTG | 32mer, [80, 47] start, [52, 48] end |
| Oligo135 | TAAAGCCCTAGGTTGGGTTATATATGGCGAAA | 32mer, [83, 48] start, [55, 47] end |
| Oligo136 | TTTTTTTTTTTTAACTGAACACTCCATGTTACTTTTTTTTTTTT | 44mer, [87, 17] start, [106, 17] end |
| Oligo137 | TTAAGCCCATATTTTCATTTGCGAAATACCGA | 32mer, [88, 47] start, [60, 48] end |
| Oligo138 | TTTTTTTTTTCGAAGCCCTTTTAGTAATCTTGACTTTTTTTTTT | 44mer, [91, 17] start, [102, 17] end |
| Oligo139 | AACGGAATCATAAAGCTAAATGTACAGTAGGG | 32mer, [92, 47] start, [64, 48] end |
| Oligo140 | TTTTTTTTTTGTTAGCAAACGTAATTTCAACTTTTTTTTTTTTT | 44mer, [95, 17] start, [98, 17] end |
| Oligo141 | GAATAGATCATACATATATTTTAAAAGCCTTTTCAGGCAG | 40mer, [96, 55] start, [66, 48] end |
| Oligo142 | CATTATACCAGTCAGGACGTTGGGACACCACG | 32mer, [97, 40] start, [96, 56] end |
| Oligo143 | TTTTTTTTTTAATCATTGTGAAACCGCCACCCTCTTTTTTTTTT | 44mer, [99, 17] start, [124, 17] end |
| Oligo144 | ATTTTGTCAGTAAATTGCCCGGAAGTCTTTCCAGAGGATA | 40mer, [99, 56] start, [122, 48] end |
| Oligo145 | GAATAAGGGTAATTAAGACTCCTTTGCGGGAG | 32mer, [101, 48] start, [73, 47] end |
| Oligo146 | GTAACAAAAAGAACTGGCATGAGACGGCGGTTGTACCAAAAACATGTA | 48mer, [100, 47] start, [65, 47] end |
| Oligo147 | GACTTGAGTAGTAAATAAGAGGCTGAGACTCCCTATTTCGGCTGACCT | 48mer, [100, 63] start, [102, 48] end |
| Oligo148 | TTTTTTTTTTAAGAACCGGATAAGGATTAGGATTTTTTTTTTTT | 44mer, [103, 17] start, [120, 17] end |
| Oligo149 | TTGAAAGATATAATGAAATAGCAAAGTAGTAG | 32mer, [105, 48] start, [77, 47] end |
| Oligo150 | AAGGGAACAAGAGCAAGAAACTCATCCGGGCGCGAGCTGAAACACCGG | 48mer, [104, 47] start, [61, 47] end |
| Oligo151 | AGCGCGTTTAATTGTATGATGATACAGGAGTGCTGAATTTGAAATCCG | 48mer, [104, 63] start, [106, 48] end |
| Oligo152 | TTTTTTTTTTAGCCGGAACGAGATAAGTTTTAACTTTTTTTTTT | 44mer, [107, 17] start, [116, 17] end |
| Oligo153 | ATTGTGTCACCGTTCCTTAAAGGCCGCTTAAA | 32mer, [106, 63] start, [114, 48] end |
| Oligo154 | AACAAAGTATACAGGGAAGCGCATTTCCCAAT | 32mer, [109, 48] start, [81, 47] end |
| Oligo155 | CCAGCGATAATAACATAAAAATCGTATTACGG | 32mer, [108, 47] start, [80, 48] end |
| Oligo156 | CCTCAGAGTGCGGGATCAGGAGGTTGAGGCAG | 32mer, [108, 63] start, [112, 48] end |
| Oligo157 | TTTTTTTTTTGAATACACTAAATTGGCCTTGATTTTTTTTTT | 42mer, [111, 17] start, [112, 17] end |
| Oligo158 | CACCAACCTAAAACGAAAGAGGCAAAATTTTTTTTTT | 37mer, [110, 55] start, [110, 17] end |
| Oligo159 | GTCAGACGAACACTCATCTTTCCAAAATAGCAGCCTTTACTATGCAAC | 48mer, [112, 47] start, [83, 47] end |
| Oligo160 | TTTTTTTTTTTATTCACAAACAAATAAATCCTCATTTCCGGACCC | 45mer, [113, 17] start, [108, 48] end |
| Oligo161 | GCCAGAATAAGCGCGACGACCTGCCCTGAACA | 32mer, [114, 47] start, [87, 47] end |
| Oligo162 | CGCAGTCTTACTGGTAGCGCAGACGGTCAATCGAATTGAGAAGTCAGA | 48mer, [115, 40] start, [87, 55] end |
| Oligo163 | TTTTTTTTTTGGGGTCAGTGCCTTGAGTAACAGTGCTTTTCATCAT | 46mer, [117, 17] start, [104, 48] end |
| Oligo164 | CCAAAAGGAGCCCCGTATAAACAGGACCAACTTCATCAAGTAAGAAAA | 48mer, [118, 63] start, [91, 47] end |
| Oligo165 | CCCCCTGCTCAAGAGATTCATTACCCAAATTTGCAATAATGTAAGCAG | 48mer, [119, 40] start, [91, 55] end |
| Oligo167 | AGTGCCGTCATTCAGTGATGGTTTAGAAAATATTTAAGAATAATGTGT | 48mer, [122, 47] start, [71, 47] end |
| Oligo168 | GGTTGATACCCTCAGATTACCTTATGCAGTTT | 32mer, [123, 40] start, [99, 55] end |
| Oligo169 | TTTTTTTTTTAGAACCGCCACCCTCAGAGCCACCACCC | 38mer, [125, 17] start, [125, 55] end |
| Oligo170 | TCATTTTCAGGTTTAGTACCGCCATAAGTATAGGGCTTGA | 40mer, [125, 56] start, [98, 48] end |
| Oligo171 | TTTTTTTTTTTTCCAGTTTGGAAGAAAATGCTGAATTTTTTTTTTT | 46mer, [0, 110] start, [27, 110] end |
| Oligo172 | TTTTTTTTTTTGCCCCAGCAGGCCGAACTGATAGCCTTTTTTTTTT | 46mer, [2, 110] start, [25, 110] end |
| Oligo173 | TTTTTTTTTTGCCCTTCACCGCCAAGAATACGTGGCTTTTTTTTTT | 46mer, [4, 110] start, [23, 110] end |
| Oligo174 | TTTTTTTTTTCATTAATGAATCGTGGATTATTTACATTTTTTTTTT | 46mer, [6, 110] start, [21, 110] end |
| Oligo175 | TTTTTTTTTTTGCGTTGCGCTCACAGGAAAAACGCTTTTTTTTTTT | 46mer, [8, 110] start, [19, 110] end |
| Oligo176 | TTTTTTTTTTAAATTGTTATCCGTTTGATTAGTAATTTTTTTTTTT | 46mer, [10, 110] start, [17, 110] end |
| Oligo177 | TTTTTTTTTTCCCGGGTACCGAGCCGAGTAAAAGAGTTTTTTTTTT | 46mer, [12, 110] start, [15, 110] end |
| Oligo178 | TGCACTTCCGAGTCACAGGAGAATGGATCTTTTTTTTTT | 39mer, [13, 72] start, [13, 110] end |
| Oligo179 | CCGTTTCTAGGTCATGGTCATAGCTGTTTCCTGTGTGTTTTTTTTTT | 47mer, [14, 79] start, [11, 110] end |
| Oligo180 | TTTTTTTTTTTCTGTCCATCACGAAACCAGCAATACTTTTTTTTTT | 46mer, [14, 110] start, [39, 110] end |
| Oligo181 | AATACTTCCTCACAATAGTGAGCTAACTCACATTAATTTTTTTTTTT | 47mer, [17, 80] start, [9, 110] end |
| Oligo182 | TTTTTTTTTTAACATCACTTGCCACTCAACGAGCAGTTTTTTTTTT | 46mer, [16, 110] start, [37, 110] end |
| Oligo183 | ACGCTGGCGTAAGTCGGGAAACCTGTCGTGCCAGCTGTTTTTTTTTT | 47mer, [18, 79] start, [7, 110] end |
| Oligo184 | TTTTTTTTTTCATGGAAATACCTCGTATTGCTAAACTTTTTTTTTT | 46mer, [18, 110] start, [35, 110] end |
| Oligo185 | GTCTGAAAGCCAACGCTGAGACGGGCAACAGCTGATTTTTTTTTTTT | 47mer, [21, 80] start, [5, 110] end |
| Oligo186 | TTTTTTTTTTTTGGCAGATTCACACTGGTGACCTGGTTTTTTTTTT | 46mer, [20, 110] start, [33, 110] end |
| Oligo187 | CTATTGAGAACGTTGCAGCAAGCGGTCCACGCTGGTTTTTTTTTTTT | 47mer, [22, 79] start, [3, 110] end |
| Oligo188 | TTTTTTTTTTACAGACAATATTTTAACTATCGACATTTTTTTTTTT | 46mer, [22, 110] start, [31, 110] end |
| Oligo189 | TTAATGCGGAAAATCCCCGAGATAGGGTTGAGTGTTGTTTTTTTTTT | 47mer, [25, 80] start, [1, 110] end |
| Oligo190 | TTTTTTTTTTCTAAAACATCGCCTTCGCATCAAAGGTTTTTTTTTT | 46mer, [24, 110] start, [29, 110] end |
| Oligo191 | TTTTTTTTTTTTCGCGTCGTCTTCACAGCGATGCCAGAGTCTGTAGT | 47mer, [26, 110] start, [26, 64] end |
| Oligo192 | TTTTTTTTTTAGAGTGAGATCGGTTCTGGTGCCGGATTTTTTTTTT | 46mer, [28, 110] start, [55, 110] end |
| Oligo193 | TTTTTTTTTTCATTACGCATCGCGAATCCTTGAAAATTTTTTTTTT | 46mer, [30, 110] start, [53, 110] end |
| Oligo194 | TTTTTTTTTTAAGAGTTTCTGCGGAGTGAATAACCTTTTTTTTTTT | 46mer, [32, 110] start, [51, 110] end |
| Oligo195 | TTTTTTTTTTTGGAAAGCAACGAGATGAAACAAACATTTTTTTTTT | 46mer, [34, 110] start, [49, 110] end |
| Oligo196 | TTTTTTTTTTCGTGAGTATTACGAAAATCGCGCAGATTTTTTTTTT | 46mer, [36, 110] start, [47, 110] end |
| Oligo197 | TTTTTTTTTTATCAAACGCCGCGTTCAGGTTTAACGTTTTTTTTTT | 46mer, [38, 110] start, [45, 110] end |
| Oligo198 | TTTTTTTTTTACCATCGATTGTGCCATATCAAAATTTTTTTTTTTT | 46mer, [40, 110] start, [43, 110] end |
| Oligo199 | GACGCAGACCTTTTCCATGAATTGGTAACTTTTTTTTTT | 39mer, [41, 72] start, [41, 110] end |
| Oligo200 | TTTTTTTTTTATTTGCACGTAAAGCCTGAGAGTCTGTTTTTTTTTT | 46mer, [42, 110] start, [67, 110] end |
| Oligo201 | TTTTTTTTTTTCAGATGAATATACAAAAACAGGAAGTTTTTTTTTT | 46mer, [44, 110] start, [65, 110] end |
| Oligo202 | TTTTTTTTTTGGCGAATTATTCATTGTTAAAATTCGTTTTTTTTTT | 46mer, [46, 110] start, [63, 110] end |
| Oligo203 | TTTTTTTTTTTCAAGAAAACAAAAGCTTTCATCAACTTTTTTTTTT | 46mer, [48, 110] start, [61, 110] end |
| Oligo204 | TTTTTTTTTTTGCTTCTGTAAATGGGAACAAACGGCTTTTTTTTTT | 46mer, [50, 110] start, [59, 110] end |
| Oligo205 | TTTTTTTTTTCATAGCGATAGCTAGTATCGGCCTCATTTTTTTTTT | 46mer, [52, 110] start, [57, 110] end |
| Oligo206 | TTTTTTTTTTAACCAGGCAAAGCGCCATTCGCCATTCAGGCTGCGCA | 47mer, [54, 110] start, [54, 64] end |
| Oligo207 | TTTTTTTTTTGGAAGATCGCACTCCTTTTGATAAGATTTTTTTTTT | 46mer, [56, 110] start, [83, 110] end |
| Oligo208 | TTTTTTTTTTGGATTGACCGTAATAACGAGCGTCTTTTTTTTTTTT | 46mer, [58, 110] start, [81, 110] end |
| Oligo209 | TTTTTTTTTTATTAAATGTGAGCTTAGTTGCTATTTTTTTTTTTTT | 46mer, [60, 110] start, [79, 110] end |
| Oligo210 | TTTTTTTTTTCATTAAATTTTTGAAATCAGATATAGTTTTTTTTTT | 46mer, [62, 110] start, [77, 110] end |
| Oligo211 | TTTTTTTTTTATTGTATAAGCAACAAGCCGTTTTTATTTTTTTTTT | 46mer, [64, 110] start, [75, 110] end |
| Oligo212 | TTTTTTTTTTGAGCAAACAAGAGTTTACGAGCATGTTTTTTTTTTT | 46mer, [66, 110] start, [73, 110] end |
| Oligo213 | TTTTTTTTTTAGCTATTTTTGAGGTTTATCAACAATTTTTTTTTTT | 46mer, [68, 110] start, [71, 110] end |
| Oligo214 | CTAGCTGATAAATTAATGCCGGAGAGGGTTTTTTTTTTT | 39mer, [69, 72] start, [69, 110] end |
| Oligo215 | TTTTTTTTTTAGATAAGTCCTGAAGGAATACCACATTTTTTTTTTT | 46mer, [70, 110] start, [95, 110] end |
| Oligo216 | TTTTTTTTTTAGAAACCAATCAAACGATAAAAACCATTTTTTTTTT | 46mer, [72, 110] start, [93, 110] end |
| Oligo217 | TTTTTTTTTTTTTTCATCGTAGGGGGGTAATAGTAATTTTTTTTTT | 46mer, [74, 110] start, [91, 110] end |
| Oligo218 | TTTTTTTTTTAAGGCTTATCCGGGAAAACGAGAATGTTTTTTTTTT | 46mer, [76, 110] start, [89, 110] end |
| Oligo219 | TTTTTTTTTTTGCACCCAGCTACAGTCAGAAGCAAATTTTTTTTTT | 46mer, [78, 110] start, [87, 110] end |
| Oligo220 | TTTTTTTTTTTCCAGAGCCTAATCGGAAGCAAACTCTTTTTTTTTT | 46mer, [80, 110] start, [85, 110] end |
| Oligo221 | TTTTTTTTTTGGTCATTTTTGCGGATGGCTTAGAGCTTA | 39mer, [82, 110] start, [82, 72] end |
| Oligo222 | TTTTTTTTTTCAACAGGTCAGGAACTTTTTCATGAGTTTTTTTTTT | 46mer, [84, 110] start, [111, 110] end |
| Oligo223 | TTTTTTTTTTGCGGATTGCATCAGCCTCCCTCAGAGTTTTTTTTTT | 46mer, [86, 110] start, [109, 110] end |
| Oligo224 | TTTTTTTTTTACCATAAATCAAATTGCCATCTTTTCTTTTTTTTTT | 46mer, [88, 110] start, [107, 110] end |
| Oligo225 | TTTTTTTTTTAATGTTTAGACTGCACCGTAATCAGTTTTTTTTTTT | 46mer, [90, 110] start, [105, 110] end |
| Oligo226 | TTTTTTTTTTAAATAGCGAGAGGCCATTACCATTAGTTTTTTTTTT | 46mer, [92, 110] start, [103, 110] end |
| Oligo227 | TTTTTTTTTTTCAACTAATGCAGGGTAAATATTGACTTTTTTTTTT | 46mer, [94, 110] start, [101, 110] end |
| Oligo228 | TTTTTTTTTTAACATTATTACAGTTTACCAGCGCCATTTTTTTTTT | 46mer, [96, 110] start, [99, 110] end |
| Oligo229 | TCTACGTTAATAAAACGAACTAACGGAACTTTTTTTTTT | 39mer, [97, 72] start, [97, 110] end |
| Oligo230 | TTTTTTTTTTAAGACAAAAGGGCGCATTCCACAGACTTTTTTTTTT | 46mer, [98, 110] start, [123, 110] end |
| Oligo231 | TTTTTTTTTTGGAAATTATTCATAAACAACTTTCAATTTTTTTTTT | 46mer, [100, 110] start, [121, 110] end |
| Oligo232 | TTTTTTTTTTCAAGGCCGGAAACGAATTGCGAATAATTTTTTTTTT | 46mer, [102, 110] start, [119, 110] end |
| Oligo233 | TTTTTTTTTTAGCGACAGAATCAGTGAATTTCTTAATTTTTTTTTT | 46mer, [104, 110] start, [117, 110] end |
| Oligo234 | TTTTTTTTTTATAATCAAAATCACCATCGCCCACGCTTTTTTTTTT | 46mer, [106, 110] start, [115, 110] end |
| Oligo235 | TTTTTTTTTTCCGCCACCCTCAGGCATCGGAACGAGTTTTTTTTTT | 46mer, [108, 110] start, [113, 110] end |
| Oligo236 | TTTTTTTTTTGAAGTTTCCATTAAACGGGTAAAATACGT | 39mer, [110, 110] start, [110, 72] end |
| Oligo237 | TTTTTTTTTTGGTAGCAACGGCTACAGAGGCTTTCCGCC | 39mer, [112, 110] start, [112, 72] end |
| Oligo238 | TTTTTTTTTTATAACCGATATATTCGGTCGCGAAAGACA | 39mer, [114, 110] start, [113, 87] end |
| Oligo239 | TTTTTTTTTTACAGCTTGATACCGATAGTTGCGCCGTACGCCCCCTT | 47mer, [116, 110] start, [107, 79] end |
| Oligo240 | TTTTTTTTTTTAATTTTTTCACGTTGAAAATCTTTCGAG | 39mer, [118, 110] start, [117, 87] end |
| Oligo241 | TTTTTTTTTTCAGTTTCAGCGGAGTGAGAATAGAAAATGAAAATCAC | 47mer, [120, 110] start, [103, 79] end |
| Oligo242 | TTTTTTTTTTAGCCCTCATAGTTAGCGTAACATTTTGCT | 39mer, [122, 110] start, [121, 87] end |
| Oligo243 | TTTTTTTTTTCACTGAGTTTCGTCACCAGTACAAACCCGTAGAAAAT | 47mer, [124, 110] start, [99, 79] end |
| Oligo244 | CAAGCCCAATAGGAACCCATGTACCGTAATTTTTTTTTT | 39mer, [125, 72] start, [125, 110] end |

**Table S4. Staple sequence replacements for assembling SQB with eight binding sites.**

For HPLC-based quantification of peptide charge–dependent non-specific binding, we assembled SQBs carrying eight binding sites. Assembly started from the 245 bare SQB staples in Table S3; 8 staples with oligo name numbers matching those listed here were replaced with the sequences provided in this table, folding the eight-site SQB. Oligo names follow Table S3, and an asterisk (*) denotes staples that were replaced.

| **Name** | **Staple sequence + Complementary ssDNA1 sequence on SQB** |
| --- | --- |
| Oligo186* | TTTTTTTTTTTTGGCAGATTCACACTGGTGACCTGGCGACTCTCATTCTCTAACAGC |
| Oligo184* | TTTTTTTTTTCATGGAAATACCTCGTATTGCTAAACCGACTCTCATTCTCTAACAGC |
| Oligo182* | TTTTTTTTTTAACATCACTTGCCACTCAACGAGCAGCGACTCTCATTCTCTAACAGC |
| Oligo196* | TTTTTTTTTTCGTGAGTATTACGAAAATCGCGCAGACGACTCTCATTCTCTAACAGC |
| Oligo195* | TTTTTTTTTTTGGAAAGCAACGAGATGAAACAAACACGACTCTCATTCTCTAACAGC |
| Oligo203* | TTTTTTTTTTTCAAGAAAACAAAAGCTTTCATCAACCGACTCTCATTCTCTAACAGC |
| Oligo202* | TTTTTTTTTTGGCGAATTATTCATTGTTAAAATTCGCGACTCTCATTCTCTAACAGC |
| Oligo201* | TTTTTTTTTTTCAGATGAATATACAAAAACAGGAAGCGACTCTCATTCTCTAACAGC |

**Table S5. Staple sequence replacements for assembling SQB with twenty binding sites.**

For confocal imaging experiments on cellular uptake of SQB-peptide and on the requirement of site-specific hybridization for functional activity, we used SQBs folded with 20 binding sites. Assembly started from the 245 bare SQB staples in Table S3; 20 staples with oligo name numbers matching those listed here were replaced with the sequences provided in this table, folding the twenty-site SQB. Oligo names follow Table S3, and an asterisk (*) denotes staples that were replaced.

| **Name** | **Staple sequence + Complementary ssDNA3 sequence on SQB** |
| --- | --- |
| Oligo82* | TATCTAAAACGTCAAAGGGCGAAAAACCGTCTATCACGTCCCCTTTTAACCCTAGAA |
| Oligo79* | AAAAGATTTGGGGTCGTGACGGGGAAAGCCGGCGAACGTCCCCTTTTAACCCTAGAA |
| Oligo66* | TTTTTTTTTTCGTGGCGAGAAATTGTCTCCATGCCGTCCCCTTTTAACCCTAGAA |
| Oligo64* | TTTTTTTTTTGGGCGATGGCCCCATAAACATTGCCGTCCCCTTTTAACCCTAGAA |
| Oligo80* | TTTTTTTTTTTGATACCGTTTAGAACCTCAAATACGTCCCCTTTTAACCCTAGAA |
| Oligo75* | TTTTTTTTTTACCGTGCAAAATAGAAACCACCAGCGTCCCCTTTTAACCCTAGAA |
| Oligo93* | TTTTTTTTTTAAGGAGCGGAATACAGAGAGGTTCCGTCCCCTTTTAACCCTAGAA |
| Oligo85* | TTTTTTTTTTTCAAACCCTCAACGCATTCACCCTCGTCCCCTTTTAACCCTAGAA |
| Oligo108* | GAAGGGCGATCGGTGCGGGCCTCTTCGCCGTCCCCTTTTAACCCTAGAA |
| Oligo106* | TTTTTTTTTTTATTACGCCAGCACTATATGTAAACGTCCCCTTTTAACCCTAGAA |
| Oligo100* | TTTTTTTTTTATCTGCCACTCAAGAATCGCCATACGTCCCCTTTTAACCCTAGAA |
| Oligo96* | TTTTTTTTTTGAAGTGAGCGAAAAGGGTGAGAAACGTCCCCTTTTAACCCTAGAA |
| Oligo116* | TTTTTTTTTTTTTAACAACGCCAACATTATGACCCGTCCCCTTTTAACCCTAGAA |
| Oligo110* | TTTTTTTTTTTGCTGATGCAAAAGTTTCATTCCACGTCCCCTTTTAACCCTAGAA |
| Oligo127* | TTTTTTTTTTAATTCTACTAATTAGCTATCTTACCGTCCCCTTTTAACCCTAGAA |
| Oligo122* | TTTTTTTTTTCTGTAATACTTTATTACGCAGTATCGTCCCCTTTTAACCCTAGAA |
| Oligo140* | TTTTTTTTTTGTTAGCAAACGTAATTTCAACTTTCGTCCCCTTTTAACCCTAGAA |
| Oligo138* | TTTTTTTTTTCGAAGCCCTTTTAGTAATCTTGACCGTCCCCTTTTAACCCTAGAA |
| Oligo148* | TTTTTTTTTTAAGAACCGGATAAGGATTAGGATTCGTCCCCTTTTAACCCTAGAA |
| Oligo143* | TTTTTTTTTTAATCATTGTGAAACCGCCACCCTCCGTCCCCTTTTAACCCTAGAA |

**Table S6. Staple sequence replacements for assembling SQB with thirty-eight binding sites.**

For Western blot experiments testing whether site-specific hybridization is required for functional activity, we compared SQB^hyb^-BDNF (stable, site-specific DNA hybridization) with SQB^ads^-BDNF (non-specific electrostatic adsorption of free peptide). We used SQBs folded with 38 binding sites. Assembly started from the 245 bare SQB staples in Table S3; 38 staples with oligo name numbers matching those listed here were replaced with the sequences provided in this table, folding the thirty-eight–site SQB. Oligo names follow Table S3, and an asterisk (*) denotes staples that were replaced.

| **Name** | **Staple sequence + Complementary ssDNA1 sequence on SQB** |
| --- | --- |
| Oligo176* | TTTTTTTTTTAAATTGTTATCCGTTTGATTAGTAATCGACTCTCATTCTCTAACAGC |
| Oligo175* | TTTTTTTTTTTGCGTTGCGCTCACAGGAAAAACGCTCGACTCTCATTCTCTAACAGC |
| Oligo174* | TTTTTTTTTTCATTAATGAATCGTGGATTATTTACACGACTCTCATTCTCTAACAGC |
| Oligo173* | TTTTTTTTTTGCCCTTCACCGCCAAGAATACGTGGCCGACTCTCATTCTCTAACAGC |
| Oligo172* | TTTTTTTTTTTGCCCCAGCAGGCCGAACTGATAGCCCGACTCTCATTCTCTAACAGC |
| Oligo190* | TTTTTTTTTTCTAAAACATCGCCTTCGCATCAAAGGCGACTCTCATTCTCTAACAGC |
| Oligo188* | TTTTTTTTTTACAGACAATATTTTAACTATCGACATCGACTCTCATTCTCTAACAGC |
| Oligo186* | TTTTTTTTTTTTGGCAGATTCACACTGGTGACCTGGCGACTCTCATTCTCTAACAGC |
| Oligo184* | TTTTTTTTTTCATGGAAATACCTCGTATTGCTAAACCGACTCTCATTCTCTAACAGC |
| Oligo182* | TTTTTTTTTTAACATCACTTGCCACTCAACGAGCAGCGACTCTCATTCTCTAACAGC |
| Oligo180* | TTTTTTTTTTTCTGTCCATCACGAAACCAGCAATACCGACTCTCATTCTCTAACAGC |
| Oligo197* | TTTTTTTTTTATCAAACGCCGCGTTCAGGTTTAACGCGACTCTCATTCTCTAACAGC |
| Oligo196* | TTTTTTTTTTCGTGAGTATTACGAAAATCGCGCAGACGACTCTCATTCTCTAACAGC |
| Oligo195* | TTTTTTTTTTTGGAAAGCAACGAGATGAAACAAACACGACTCTCATTCTCTAACAGC |
| Oligo194* | TTTTTTTTTTAAGAGTTTCTGCGGAGTGAATAACCTCGACTCTCATTCTCTAACAGC |
| Oligo193* | TTTTTTTTTTCATTACGCATCGCGAATCCTTGAAAACGACTCTCATTCTCTAACAGC |
| Oligo205* | TTTTTTTTTTCATAGCGATAGCTAGTATCGGCCTCACGACTCTCATTCTCTAACAGC |
| Oligo204* | TTTTTTTTTTTGCTTCTGTAAATGGGAACAAACGGCCGACTCTCATTCTCTAACAGC |
| Oligo203* | TTTTTTTTTTTCAAGAAAACAAAAGCTTTCATCAACCGACTCTCATTCTCTAACAGC |
| Oligo202* | TTTTTTTTTTGGCGAATTATTCATTGTTAAAATTCGCGACTCTCATTCTCTAACAGC |
| Oligo201* | TTTTTTTTTTTCAGATGAATATACAAAAACAGGAAGCGACTCTCATTCTCTAACAGC |
| Oligo200* | TTTTTTTTTTATTTGCACGTAAAGCCTGAGAGTCTGCGACTCTCATTCTCTAACAGC |
| Oligo212* | TTTTTTTTTTGAGCAAACAAGAGTTTACGAGCATGTCGACTCTCATTCTCTAACAGC |
| Oligo211* | TTTTTTTTTTATTGTATAAGCAACAAGCCGTTTTTACGACTCTCATTCTCTAACAGC |
| Oligo210* | TTTTTTTTTTCATTAAATTTTTGAAATCAGATATAGCGACTCTCATTCTCTAACAGC |
| Oligo209* | TTTTTTTTTTATTAAATGTGAGCTTAGTTGCTATTTCGACTCTCATTCTCTAACAGC |
| Oligo208* | TTTTTTTTTTGGATTGACCGTAATAACGAGCGTCTTCGACTCTCATTCTCTAACAGC |
| Oligo220* | TTTTTTTTTTTCCAGAGCCTAATCGGAAGCAAACTCCGACTCTCATTCTCTAACAGC |
| Oligo219* | TTTTTTTTTTTGCACCCAGCTACAGTCAGAAGCAAACGACTCTCATTCTCTAACAGC |
| Oligo218* | TTTTTTTTTTAAGGCTTATCCGGGAAAACGAGAATGCGACTCTCATTCTCTAACAGC |
| Oligo217* | TTTTTTTTTTTTTTCATCGTAGGGGGGTAATAGTAACGACTCTCATTCTCTAACAGC |
| Oligo216* | TTTTTTTTTTAGAAACCAATCAAACGATAAAAACCACGACTCTCATTCTCTAACAGC |
| Oligo215* | TTTTTTTTTTAGATAAGTCCTGAAGGAATACCACATCGACTCTCATTCTCTAACAGC |
| Oligo227* | TTTTTTTTTTTCAACTAATGCAGGGTAAATATTGACCGACTCTCATTCTCTAACAGC |
| Oligo226* | TTTTTTTTTTAAATAGCGAGAGGCCATTACCATTAGCGACTCTCATTCTCTAACAGC |
| Oligo225* | TTTTTTTTTTAATGTTTAGACTGCACCGTAATCAGTCGACTCTCATTCTCTAACAGC |
| Oligo224* | TTTTTTTTTTACCATAAATCAAATTGCCATCTTTTCCGACTCTCATTCTCTAACAGC |
| Oligo223* | TTTTTTTTTTGCGGATTGCATCAGCCTCCCTCAGAGCGACTCTCATTCTCTAACAGC |

**Table S7. Staple sequence replacements for assembling SQB with twenty-four binding sites for FITC (24:0).**

For confocal imaging experiments on cellular uptake of SQB-peptide, we used SQBs folded with 24 binding sites to conjugate the FITC-ssDNA1. Assembly started from the 245 bare SQB staples in Table S3; 24 staples with oligo name numbers matching those listed here were replaced with the sequences provided in this table, folding the twenty-four fluorescent dye site SQB. Oligo names follow Table S3, and an asterisk (*) denotes staples that were replaced.

| **Name** | **Staple sequence + Complementary ssDNA1 sequence on SQB** |
| --- | --- |
| Oligo175* | TTTTTTTTTTTGCGTTGCGCTCACAGGAAAAACGCTCGACTCTCATTCTCTAACAGC |
| Oligo174* | TTTTTTTTTTCATTAATGAATCGTGGATTATTTACACGACTCTCATTCTCTAACAGC |
| Oligo173* | TTTTTTTTTTGCCCTTCACCGCCAAGAATACGTGGCCGACTCTCATTCTCTAACAGC |
| Oligo172* | TTTTTTTTTTTGCCCCAGCAGGCCGAACTGATAGCCCGACTCTCATTCTCTAACAGC |
| Oligo188* | TTTTTTTTTTACAGACAATATTTTAACTATCGACATCGACTCTCATTCTCTAACAGC |
| Oligo186* | TTTTTTTTTTTTGGCAGATTCACACTGGTGACCTGGCGACTCTCATTCTCTAACAGC |
| Oligo184* | TTTTTTTTTTCATGGAAATACCTCGTATTGCTAAACCGACTCTCATTCTCTAACAGC |
| Oligo182* | TTTTTTTTTTAACATCACTTGCCACTCAACGAGCAGCGACTCTCATTCTCTAACAGC |
| Oligo196* | TTTTTTTTTTCGTGAGTATTACGAAAATCGCGCAGACGACTCTCATTCTCTAACAGC |
| Oligo195* | TTTTTTTTTTTGGAAAGCAACGAGATGAAACAAACACGACTCTCATTCTCTAACAGC |
| Oligo194* | TTTTTTTTTTAAGAGTTTCTGCGGAGTGAATAACCTCGACTCTCATTCTCTAACAGC |
| Oligo193* | TTTTTTTTTTCATTACGCATCGCGAATCCTTGAAAACGACTCTCATTCTCTAACAGC |
| Oligo204* | TTTTTTTTTTTGCTTCTGTAAATGGGAACAAACGGCCGACTCTCATTCTCTAACAGC |
| Oligo203* | TTTTTTTTTTTCAAGAAAACAAAAGCTTTCATCAACCGACTCTCATTCTCTAACAGC |
| Oligo202* | TTTTTTTTTTGGCGAATTATTCATTGTTAAAATTCGCGACTCTCATTCTCTAACAGC |
| Oligo201* | TTTTTTTTTTTCAGATGAATATACAAAAACAGGAAGCGACTCTCATTCTCTAACAGC |
| Oligo211* | TTTTTTTTTTATTGTATAAGCAACAAGCCGTTTTTACGACTCTCATTCTCTAACAGC |
| Oligo210* | TTTTTTTTTTCATTAAATTTTTGAAATCAGATATAGCGACTCTCATTCTCTAACAGC |
| Oligo209* | TTTTTTTTTTATTAAATGTGAGCTTAGTTGCTATTTCGACTCTCATTCTCTAACAGC |
| Oligo208* | TTTTTTTTTTGGATTGACCGTAATAACGAGCGTCTTCGACTCTCATTCTCTAACAGC |
| Oligo219* | TTTTTTTTTTTGCACCCAGCTACAGTCAGAAGCAAACGACTCTCATTCTCTAACAGC |
| Oligo218* | TTTTTTTTTTAAGGCTTATCCGGGAAAACGAGAATGCGACTCTCATTCTCTAACAGC |
| Oligo217* | TTTTTTTTTTTTTTCATCGTAGGGGGGTAATAGTAACGACTCTCATTCTCTAACAGC |
| Oligo216* | TTTTTTTTTTAGAAACCAATCAAACGATAAAAACCACGACTCTCATTCTCTAACAGC |

**Table S8. Staple sequence replacements for assembling SQB with twenty-four binding sites for FITC and Cy5.5 (12:12).**

For confocal imaging experiments on cellular uptake of SQB-peptide, we used SQBs folded with 24 binding sites to conjugate the FITC-ssDNA1 and Cy5.5-ssDNA2. Assembly started from the 245 bare SQB staples in Table S3; 24 staples with oligo name numbers matching those listed here were replaced with the sequences provided in this table, folding the twenty-four fluorescent dye site SQB. Oligo names follow Table S3, and an asterisk (*) denotes staples that were replaced.

| **Name** | **Staple sequence + Complementary ssDNA1/2 sequence on SQB** |
| --- | --- |
| Oligo175* | TTTTTTTTTTTGCGTTGCGCTCACAGGAAAAACGCTCGACTCTCATTCTCTAACAGC |
| Oligo174* | TTTTTTTTTTCATTAATGAATCGTGGATTATTTACACGACTCTCATTCTCTAACAGC |
| Oligo173* | TTTTTTTTTTGCCCTTCACCGCCAAGAATACGTGGCCTCACATGGTCTCACATCACT |
| Oligo172* | TTTTTTTTTTTGCCCCAGCAGGCCGAACTGATAGCCCTCACATGGTCTCACATCACT |
| Oligo188* | TTTTTTTTTTACAGACAATATTTTAACTATCGACATCTCACATGGTCTCACATCACT |
| Oligo186* | TTTTTTTTTTTTGGCAGATTCACACTGGTGACCTGGCTCACATGGTCTCACATCACT |
| Oligo184* | TTTTTTTTTTCATGGAAATACCTCGTATTGCTAAACCGACTCTCATTCTCTAACAGC |
| Oligo182* | TTTTTTTTTTAACATCACTTGCCACTCAACGAGCAGCGACTCTCATTCTCTAACAGC |
| Oligo196* | TTTTTTTTTTCGTGAGTATTACGAAAATCGCGCAGACGACTCTCATTCTCTAACAGC |
| Oligo195* | TTTTTTTTTTTGGAAAGCAACGAGATGAAACAAACACGACTCTCATTCTCTAACAGC |
| Oligo194* | TTTTTTTTTTAAGAGTTTCTGCGGAGTGAATAACCTCTCACATGGTCTCACATCACT |
| Oligo193* | TTTTTTTTTTCATTACGCATCGCGAATCCTTGAAAACTCACATGGTCTCACATCACT |
| Oligo204* | TTTTTTTTTTTGCTTCTGTAAATGGGAACAAACGGCCTCACATGGTCTCACATCACT |
| Oligo203* | TTTTTTTTTTTCAAGAAAACAAAAGCTTTCATCAACCTCACATGGTCTCACATCACT |
| Oligo202* | TTTTTTTTTTGGCGAATTATTCATTGTTAAAATTCGCGACTCTCATTCTCTAACAGC |
| Oligo201* | TTTTTTTTTTTCAGATGAATATACAAAAACAGGAAGCGACTCTCATTCTCTAACAGC |
| Oligo211* | TTTTTTTTTTATTGTATAAGCAACAAGCCGTTTTTACGACTCTCATTCTCTAACAGC |
| Oligo210* | TTTTTTTTTTCATTAAATTTTTGAAATCAGATATAGCGACTCTCATTCTCTAACAGC |
| Oligo209* | TTTTTTTTTTATTAAATGTGAGCTTAGTTGCTATTTCTCACATGGTCTCACATCACT |
| Oligo208* | TTTTTTTTTTGGATTGACCGTAATAACGAGCGTCTTCTCACATGGTCTCACATCACT |
| Oligo219* | TTTTTTTTTTTGCACCCAGCTACAGTCAGAAGCAAACTCACATGGTCTCACATCACT |
| Oligo218* | TTTTTTTTTTAAGGCTTATCCGGGAAAACGAGAATGCTCACATGGTCTCACATCACT |
| Oligo217* | TTTTTTTTTTTTTTCATCGTAGGGGGGTAATAGTAACGACTCTCATTCTCTAACAGC |
| Oligo216* | TTTTTTTTTTAGAAACCAATCAAACGATAAAAACCACGACTCTCATTCTCTAACAGC |

**Table S9. Staple sequence replacements for assembling SQB with twenty-four binding sites for Cy5.5 (0:24).**

For confocal imaging experiments on cellular uptake of SQB-peptide, we used SQBs folded with 24 binding sites to conjugate the Cy5.5-ssDNA2. Assembly started from the 245 bare SQB staples in Table S3; 24 staples with oligo name numbers matching those listed here were replaced with the sequences provided in this table, folding the twenty-four fluorescent dye site SQB. Oligo names follow Table S3, and an asterisk (*) denotes staples that were replaced.

| **Name** | **Staple sequence + Complementary ssDNA2 sequence on SQB** |
| --- | --- |
| Oligo175* | TTTTTTTTTTTGCGTTGCGCTCACAGGAAAAACGCTCTCACATGGTCTCACATCACT |
| Oligo174* | TTTTTTTTTTCATTAATGAATCGTGGATTATTTACACTCACATGGTCTCACATCACT |
| Oligo173* | TTTTTTTTTTGCCCTTCACCGCCAAGAATACGTGGCCTCACATGGTCTCACATCACT |
| Oligo172* | TTTTTTTTTTTGCCCCAGCAGGCCGAACTGATAGCCCTCACATGGTCTCACATCACT |
| Oligo188* | TTTTTTTTTTACAGACAATATTTTAACTATCGACATCTCACATGGTCTCACATCACT |
| Oligo186* | TTTTTTTTTTTTGGCAGATTCACACTGGTGACCTGGCTCACATGGTCTCACATCACT |
| Oligo184* | TTTTTTTTTTCATGGAAATACCTCGTATTGCTAAACCTCACATGGTCTCACATCACT |
| Oligo182* | TTTTTTTTTTAACATCACTTGCCACTCAACGAGCAGCTCACATGGTCTCACATCACT |
| Oligo196* | TTTTTTTTTTCGTGAGTATTACGAAAATCGCGCAGACTCACATGGTCTCACATCACT |
| Oligo195* | TTTTTTTTTTTGGAAAGCAACGAGATGAAACAAACACTCACATGGTCTCACATCACT |
| Oligo194* | TTTTTTTTTTAAGAGTTTCTGCGGAGTGAATAACCTCTCACATGGTCTCACATCACT |
| Oligo193* | TTTTTTTTTTCATTACGCATCGCGAATCCTTGAAAACTCACATGGTCTCACATCACT |
| Oligo204* | TTTTTTTTTTTGCTTCTGTAAATGGGAACAAACGGCCTCACATGGTCTCACATCACT |
| Oligo203* | TTTTTTTTTTTCAAGAAAACAAAAGCTTTCATCAACCTCACATGGTCTCACATCACT |
| Oligo202* | TTTTTTTTTTGGCGAATTATTCATTGTTAAAATTCGCTCACATGGTCTCACATCACT |
| Oligo201* | TTTTTTTTTTTCAGATGAATATACAAAAACAGGAAGCTCACATGGTCTCACATCACT |
| Oligo211* | TTTTTTTTTTATTGTATAAGCAACAAGCCGTTTTTACTCACATGGTCTCACATCACT |
| Oligo210* | TTTTTTTTTTCATTAAATTTTTGAAATCAGATATAGCTCACATGGTCTCACATCACT |
| Oligo209* | TTTTTTTTTTATTAAATGTGAGCTTAGTTGCTATTTCTCACATGGTCTCACATCACT |
| Oligo208* | TTTTTTTTTTGGATTGACCGTAATAACGAGCGTCTTCTCACATGGTCTCACATCACT |
| Oligo219* | TTTTTTTTTTTGCACCCAGCTACAGTCAGAAGCAAACTCACATGGTCTCACATCACT |
| Oligo218* | TTTTTTTTTTAAGGCTTATCCGGGAAAACGAGAATGCTCACATGGTCTCACATCACT |
| Oligo217* | TTTTTTTTTTTTTTCATCGTAGGGGGGTAATAGTAACTCACATGGTCTCACATCACT |
| Oligo216* | TTTTTTTTTTAGAAACCAATCAAACGATAAAAACCACTCACATGGTCTCACATCACT |
